# Mechanisms of antiviral action and toxicities of ipecac alkaloids: Emetine and dehydroemetine exhibit anti-coronaviral activities at non-cardiotoxic concentrations

**DOI:** 10.1016/j.virusres.2024.199322

**Published:** 2024-01-19

**Authors:** Viktoriya S. Sidorenko, Ira Cohen, Kunchok Dorjee, Conceição A. Minetti, David P. Remeta, Junyuan Gao, Irina Potapova, Hong Zhan Wang, Janet Hearing, Wan-Yi Yen, Hwan Keun Kim, Keiji Hashimoto, Masaaki Moriya, Kathleen G. Dickman, Xingyu Yin, Miguel Garcia-Diaz, Rajesh Chennamshetti, Radha Bonala, Francis Johnson, Amanda L. Waldeck, Ramesh Gupta, Chaoping Li, Kenneth J. Breslauer, Arthur P. Grollman, Thomas A. Rosenquist

**Affiliations:** a Department of Pharmacological Sciences, Renaissance School of Medicine, Stony Brook University, Stony Brook, NY 11794, USA; b Department of Physiology, Renaissance School of Medicine, Stony Brook University, Stony Brook, New York 11794, USA; c Division of Infectious Diseases, John Hopkins School of Medicine, Baltimore, Maryland 21205, USA; d Department of Chemistry and Chemical Biology, Rutgers – The State University of New Jersey, Piscataway, New Jersey 08854, USA; e Department of Microbiology and Immunology, Renaissance School of Medicine, Stony Brook University, Stony Brook, New York 11794, USA; f Department of Medicine, Renaissance School of Medicine, Stony Brook University, Stony Brook, New York 11794, USA; g Department of Chemistry, Stony Brook University, Stony Brook, New York 11794, USA; h Department of Pharmacy, Stony Brook University Hospital, Stony Brook, New York 11794, USA; i ChemMaster International Inc., Happauge, New York 11788, USA; j Chemistry Service Unit of Shanghai Haoyuan Chemexpress Co., Ltd., Shanghai, PR China 201203; k Rutgers Cancer Institute of New Jersey, New Brunswick, NJ 08901, USA

**Keywords:** Emetine, Dehydroemetine, SARS-CoV-2, Coronoviruses, Antiviral

## Abstract

The emergence of highly infectious pathogens with their potential for triggering global pandemics necessitate the development of effective treatment strategies, including broad-spectrum antiviral therapies to safeguard human health. This study investigates the antiviral activity of emetine, dehydroemetine (DHE), and congeneric compounds against SARS-CoV-2 and HCoV-OC43, and evaluates their impact on the host cell. Concurrently, we assess the potential cardiotoxicity of these ipecac alkaloids. Significantly, our data reveal that emetine and the (-)-R,S isomer of 2,3-dehydroemetine (designated in this paper as DHE4) reduce viral growth at nanomolar concentrations (i.e., IC_50_ ∼ 50–100 nM), paralleling those required for inhibition of protein synthesis, while calcium channel blocking activity occurs at elevated concentrations (i.e., IC_50_ ∼ 40–60 µM). Our findings suggest that the antiviral mechanisms primarily involve disruption of host cell protein synthesis and is demonstrably stereoisomer specific. The prospect of a therapeutic window in which emetine or DHE4 inhibit viral propagation without cardiotoxicity renders these alkaloids viable candidates in strategies worthy of clinical investigation.

## Highlights

• Emetine and dehydroemetine inhibit both coronavirus growth and host-cell protein synthesis at nanomolar concentrations.
• The stereochemical requirements of emetine and its analogs for inhibition of both viral growth and host-cell protein synthesis inhibition are the same.
• Cardiotoxic effects of emetine and dehydroemetine at I_CaL_ are not stereo-specific and require micromolar concentrations.

## 1 Introduction

Outbreaks of the Middle East Respiratory (MERS) (Raj et al., 2014) and Severe Acute Respiratory (SARS and COVID-19) (Peiris et al., 2003; Wang et al., 2020b; Zhu et al., 2020; Wu et al., 2020) Syndromes, initiated by MERS-CoV, SARS-CoV, and SARS-CoV-2 coronaviruses, pose a significant public health threat and economic burden worldwide. Vaccines have failed to fully prevent the emergence and spread of SARS-CoV-2 variants, thus additional measures are required to reduce hospitalization and mortality (Wu et al., 2023). Antiviral treatment options against COVID-19 are limited and evidence of the efficacy of available antiviral treatments could not be reproduced consistently in recent clinical studies (Abdool Karim and Devnarain, 2022). These considerations have generated interest in exploring the therapeutic efficacy of small molecules like emetine, a natural alkaloid used in primarily as an amebicide (Grollman and Jarkovsky, 1975) but recently investigated for its antiviral properties towards SARS-CoV-2 (Choy et al., 2020; Bojkova et al., 2020; Jan et al., 2021; Wang et al., 2020a). Considering its unique mechanisms of action, emetine may fulfill the demand for a drug with broad-range antiviral activity and potential for synergism in combination regimens (Valipour, 2022; Bleasel and Peterson, 2020b; Bleasel and Peterson, 2020a).

**Fig. 1:**
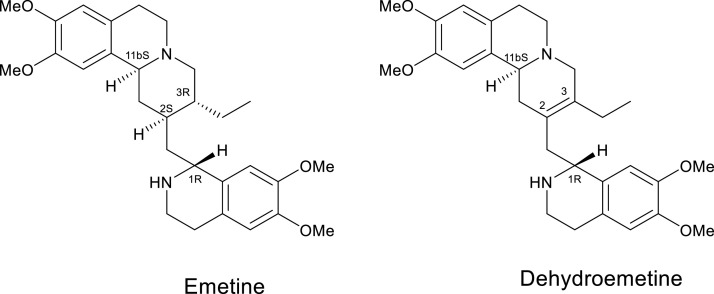
Emetine, a natural product of *Ipecachuanha* plants, and its synthetic analog dehydroemetine. While there are 16 possible diastereomers of emetine and 4 of dehydroemetine, shown are the compounds active against ameba have the 1R, 11bS, 2S, 3R- and 1R, 11bS- configurations for emetine and dehydroemetine, respectively. Emetine isolated from the plants is predominantly in the form of 1R, 11bS, 2S, 3R.

A natural product of *Cephaelia ipecachuanha* plants, emetine and its analogs (Fig. 1) have been used clinically as anti-amoebic agents for decades (Powell, 1971), and as an antiviral agents against herpes zoster (Girard et al., 1954; Grosz, 1964; Annamalai, 1965; Viegas and Viegas, 1957), influenza type A virus in the 1918 Spanish flu pandemic (Points, 1920) and viral hepatitis (Kosina and Truksova, 1976; Del Puerto et al., 1968; Fusillo, 1973). However, evidence of toxicity to the heart, muscle tissue and gastrointestinal tract (Klatskin and Friedman, 1948; Sodeman et al., 1952; Panettiere and Coltman, 1971) discouraged their use leading to their replacement by metronidazole (Knight, 1980). Subsequently, no effort has been made to clinically revive the drug. However, the various toxicities, such as cardiotoxicity and nausea, have been associated with the use of emetine at high doses (>20 mg per person per day).

Notably, there is ample evidence that emetine has broad antiviral activity at nanomolar concentrations against Zika and Ebola viruses (Yang et al., 2018), Rift Valley fever virus, influenza A virus, herpes simplex virus 2 (Andersen et al., 2019), cytomegalovirus (CMV) (Mukhopadhyay et al., 2016), and enteroviruses (Tang et al., 2020b; Tang et al., 2020a). Among coronaviruses, emetine has shown antiviral activity against hCoV-NL63, hCoV-OC43, MERS-CoV, and SARS-CoV (Shen et al., 2019), and SARS-CoV-2 (Choy et al., 2020; Bojkova et al., 2020; Jan et al., 2021; Wang et al., 2020a). It is noteworthy that emetine is at least 200-fold more potent as an antiviral than as an antiprotozoal agent in cell culture models (Burchard and Mirelman, 1988; Bleasel and Peterson, 2020b). With respect to its in vivo activity, preclinical studies have demonstrated the effectiveness of emetine against CMV (Mukhopadhyay et al., 2016), Zika virus (Yang et al., 2018) and enterovirus-71 (Tang et al., 2020a). When administered subcutaneously at 1/10th of the dose adopted for amebiasis, emetine effects are not observed in the electrocardiogram (EKG) of patients with viral hepatitis (Kosina and Truksova, 1976) or herpes zoster (Annamalai, 1965). Synergism between emetine and antiviral drugs such as ganciclovir (Mukhopadhyay et al., 2016), remdesevir (Choy et al., 2020) and dibucaine (Tang et al., 2020b) has been reported in vitro and in vivo. The synthetic analog, dehydroemetine (DHE), elicits lower cardiotoxicity than emetine (Dempsey and Salem, 1966), presumably due to a shorter half-life in heart tissues (Schwartz and Herrero, 1965). Due to its reduced cardiotoxicity, potent activity against *Plasmodium* species, and its in vitro synergism with chloroquine (Wong et al., 2014), atovaquone, and proguanil (Panwar et al., 2020) in vitro*,* DHE has been considered for the treatment of malaria (Wong et al., 2014; Panwar et al., 2020). The effectiveness of DHE as an antiviral agent for the treatment of coronavirus diseases is yet to be established.

Whereas the antiprotozoal action of emetine is primarily attributed to inhibition of eukaryotic protein synthesis elongation (Entner and Grollman, 1973), numerous mechanisms of action have been proposed for its antiviral activities including inhibition of viral RNA-dependent RNA polymerases (RdRp) (Gurung et al., 2021; Jan et al., 2021), viral entry (Yang et al., 2018; Shen et al., 2019), lysosome-mediated biogenesis of viral particles (Choy et al., 2020; Yang et al., 2018) and viral protein synthesis on mammalian ribosomes (Kumar et al., 2021; Grollman, 1968). A mechanism involving disruption of p53-MDM2 interactions mediated by the ribosomal protein S14 (RPS14) has been proposed for its antiviral properties against CMV virus (Mukhopadhyay et al., 2016). Elucidation of emetine's antiviral targets will facilitate development of therapeutics with improved safety and ADME.

In this paper we report on diastereomers of emetine and dehydroemetine with respect to their anti-coronavirus activities, and cardiotoxicity in mammalian cell culture models, potential inhibitors of host-cell protein synthesis, and blockage of the l-type calcium current I_CaL_.

## 2 Materials and methods

### 2.1 Chemicals and other materials

The sources and structures of ipecac alkaloids are shown in Supplementary Tables S1 and S2. Briefly, emetine dihydrochloride was purchased from Millipore Sigma (Calbiochem™ Sigma, MO, USA; 324,693–250MG, lot #3,511,617). Four diastereomers of dehydroemetine (DHE1, DHE2, DHE3 and DHE4) were obtained from MedChemExpress (NJ, USA); the manufacturer separated dehydroemetine by chiral HPLC and products were designated DHE1 through DHE4 in the order of elution. DHE1 and DHE4 were unambiguously identified by NMR; the remaining two diastereomers are designated DHE2 and DHE3 in this study as detailed in Supplementary Tables S1 and S2. Isoemetine dihydrochloride was synthesized by Chem-Master International Inc. (NY, USA). The quality and purity of samples were verified in our laboratories by high performance liquid chromatography, nuclear magnetic resonance (H^1^-NMR), liquid chromatography/tandem mass spectrometry and circular dichroism (CD). Stock solutions of ipecac alkaloids were prepared in DMSO at 30 mM (isoemetine), 100 mM (dehydroemetines) or 200 mM (emetine) and stored at −80 °C.

Cycloheximide (CHX), TWEEN20, trichloroacetic acid (TCA), sodium dodecyl sulfate (SDS), trypsin-EDTA, tris-buffered saline, Dulbecco's phosphate-buffered saline (PBS), methanol, puromycin and film for chemoluminescence detection were obtained from Millipore Sigma (MO, USA, Sigma). Unless indicated otherwise, reagents and equipment for electrophoresis and immunobloting were purchased from BioRad (CA, USA). Sources of other reagents are noted in descriptions of individual methods. All reagents were of ACS quality.

### 2.2 Cell lines and cell culture

African green monkey kidney cells (Vero E6) were purchased from the American Type Culture Collection (ATCC, VA, USA). The generation and characterization of human bronchial epithelial cells BEC-hACE2 were recently described (Sohn et al., 2021). Unless indicated otherwise, cell lines were maintained under standard culture conditions in a humidified incubator in 5 % CO_2_ at 37 °C. Vero E6 cells were cultured in Eagles's Minimum Essential Medium (EMEM) supplemented with 5 % fetal bovine serum (5 % FBS-EMEM). BEC-hACE2 cells were maintained in airway epithelial cell basal medium (ATCC; PCS-300–03) supplemented with bronchial epithelial cell growth kit reagents (ATCC; PCS-300–040).

### 2.3 Viruses

All in vitro experiments that involved handling SARS-CoV-2 virus were conducted in a biosafety level 3 facility located in the Center for Infectious Disease (CID) at Stony Brook University. Protocols were approved by the Stony Brook University CID Safety Committee. HCoV-OC43 experiments and isolation of viral RNA were conducted in BSL2 laboratories at the Department of Pharmacological Sciences at Stony Brook University.

SARS-CoV-2 USA-WA1/2020 was obtained from the Biodefense and Emerging Infections Research Resources Repository (BEI Resources, VA, USA; NR no. 52,281). Virus stocks were prepared by passage of the seed virus (passage 1) in Vero E6 cells at a multiplicity of infection of 0.01 per cell at 37 °C in a humidified atmosphere with 5 % CO_2_. Supernatants collected at 72 h post-infection (hpi) were clarified by centrifugation at 400 g for 10 min and then stored at −70 °C.

HCoV-OC43 was obtained from ATCC (VR-1558). Stocks were produced by infecting Vero E6 with HCoV-OC43 in 2 % FBS-EMEM at 33 °C in a 5 % CO_2_ atmosphere for 2 h with gentle mixing. Infection medium was replaced with 2 % FBS-EMEM and cells were maintained for several days. Viral stock (8.0 × 10^7^ copies/µL) was obtained by collecting and freezing supernatant from infected cells. Viral genome copy number was determined by reverse transcription coupled with quantitative PCR (RT-qPCR) using Quantitative Genomic RNA from Betacoronavirus 1 Strain OC43 (ATCC; VR-1558DQ) as a standard.

### 2.4 Inhibition of SARS-CoV-2 RNA-dependent RNA polymerase active complex

Non-structural proteins 12, 7 and 8 (Nsp12, 7 and 8) of SARS-CoV-2 were purified as described previously (Hillen et al., 2020). The RNA extension assay was performed in a reaction buffer containing 50 mM KCl, 100 mM Tris–HCl (pH 8.0) and 1 mM DTT. A Cy3 fluorescent-labeled RNA primer (5′-Cy3-GUCAUUCUCCUAAGAAGCUA-3′) was annealed to a 40 nt RNA template (5′-CUAUCCCCAUGUGAUUUUAAUAGCUUCUUAGGAGAAUGAC-3′) by heating to 75 °C and gradually cooling to 4 °C, to generate a double-strand RNA substrate. SARS-CoV-2 RdRp (nsp12) was incubated with its co-factors, nsp7 and nsp8 (1:2:2 molar ratio) on ice for 20 min prior to the extension reaction. The reactions (20μl) contained 15 mM MgCl_2_, 500 nM RNA substrate, 500 μM NTPs and varying concentrations of emetine, and were initiated by the addition of 1 μM pre-incubated RdRp complex at 37 °C. The reactions were terminated after 60 min by adding 10 μl of stop solution (95 % formamide, 20 mM EDTA). The products were separated by electrophoresis in a 16 % denaturing polyacrylamide gel and visualized using a Typhoon Imager.

### 2.5 Inhibition of protein synthesis in rabbit reticulocyte lysates

The TnT® Quick Coupled Transcription/Translation System from Promega (WI, USA; L1170) was used to evaluate inhibitory effects of ipecac alkaloids and cycloheximide on protein synthesis. Assay conditions were employed as recommended by the manufacturer. Briefly, reactions (20 μl) contained 80 % TNT Quick master mix, 20 µM methionine, 40 ng/µL luciferase RNA (Promega; L4561), and test chemicals. Samples were incubated at 30 °C for 90 min and diluted 10-fold with PBS. An aliquot (2 μl) was mixed with 20 μl of luciferase assay reagent (Promega; E1500) and luminescence was measured with a Turner Designs luminometer (model TD-20/20).

### 2.6 Inhibition of protein synthesis in cultured cells

#### 2.6.1 Puromycin pulse labeling

Vero E6 cells were seeded at 30,000 cells per cm^2^ in 6-well plates. When cells achieved ∼70–80 % maximal density two days after plating, medium was replaced with 2 % FBS-EMEM containing emetine or an analog at 0.03–10 μM and plates were incubated for one hour followed by addition of puromycin (0.01 mg/ml final concentration) as described (Dadehbeigi and Dickson, 2013). Control experiments were conducted in medium alone and medium containing cycloheximide or DMSO. After 12 min, cells were washed with 37° PBS and then allowed to recover in drug-free 2 % FBS-EMEM for 30 min. Cell pellets, collected by trypsinization and centrifugation for 5 min at 5 000 rpm at 4 °C, were re-suspended in ice-cold PBS and centrifugation was repeated. Collected cells were stored at −80 °C. Inhibition of protein synthesis in BEC-hACE2 cells by emetine and isoemetine was evaluated in a similar manner using standard culture medium for this cell line.

#### 2.6.2 Protein preparation and immunoblotting to detect puromycilated proteins

Antibodies used were anti-puromycin monoclonal (TFS; MABE34), goat anti-mouse-HRP (TFS, Invitrogen; G21040), rabbit anti-β-actin (Cell Signaling, 4967S, lot 9) and HRP-conjugated goat-anti-rabbit (Cell Signaling, 7074S, lot 27). Protein extract and immunoblotting methods were done by standard methods reported here as Supplementary Information.

### 2.7 Antiviral activity of ipecac alkaloids

#### 2.7.1 HCoV-OC34

Vero E6 cells (∼5 × 10^4^ cells/cm^2^ in 24-well plates) were infected with HCoV-OC34 at MOI of 20 genomes per cell in 2 % FBS-EMEM at 33 °C, 5 % CO2 with gently mixing for 2 h. Infection medium was removed, cells were washed with fresh medium followed by the addition of compounds at indicated concentrations in 2 % FBS-EMEM. After 72 h, 100 µL of medium was collected and stored at −80 °C for viral RNA isolation and RT-qPCR analysis as detailed below. Drugs were added to the medium prior to and after viral absorption for comparison.

#### 2.7.2 SARS-CoV-2

BEC-hACE2 cells were seeded in 12-well cell culture plates at 6–8 × 10^4^ cells per well and incubated for 2 or 3 days to obtain sub-confluent (70–80 % density) or confluent monolayers, respectively. Cells were infected at the indicated multiplicities of infection with SARS-CoV-2 virus diluted in 0.16 mL growth medium for 1 h at 37 °C with occasional rocking. After washing to remove unbound virus, 0.3 ml of growth medium containing varying concentrations of emetine, or an analog, was added. After 24 h, 140 µL of medium was collected and stored at −80 °C until RNA isolation and RT-qPCR analysis as below. Some experiments were conducted with drugs included in the medium prior to and after viral absorption.

#### 2.7.3 Isolation of viral RNA and RT-qPCR analysis

HCoV-OC43 RNA was isolated from 100 µL of medium using Quick-RNA Viral Kit-DX (Zymo Research, CA, USA; R1035). The relative amount of viral genomic RNA was determined with Luna Universal One-Step RT-qPCR Kit (New England Biolabs, MA, USA; E3005). The primers for the *N*-gene were as follows (van Elden et al., 2004):

NOC43–1: 5′-CGATGAGGCTATTCCGACTAGGT-3′.
NOC43–2: 5′-CCTTCCTGAGCCTTCAATATAGTAACC-3′.

RT-qPCR was conducted using the DNA Engine Opticon 2 System (Bio-Rad) with the following PCR cycles: 55 °C for 20 min, followed by 1 min at 95 °C and 45 cycles of 95 °C for 10 s, 53 °C for 30 s and 60 °C for 1 min.

SARS-CoV-2 RNA was prepared from 140 µL of medium using QIAmp Viral RNA Mini kit (QIAGEN, MD, USA; 52,906). The number of copies of genomic RNA was determined as described above using the following primer set (https://www.cdc.gov/coronavirus/2019-ncov/lab/rt-pcr-panel-primer-probes.html):

2019-nCoV_N1-F: 5′- GACCCCAAAATCAGCGAAAT-3′.
2019-nCoV_N1-R: 5′- TCTGGTTACTGCCAGTTGAATCTG-3′.

RT-qPCR cycles were set for 20 min at 55 °C, 1 min at 95 °C followed by 45 cycles of 95 °C for 10 s, 48 °C for 30 s and 60 °C for 1 min. The threshold cycle (Ct) for each sample was determined using the Opticon Monitor 3 Program (BioRad). Drug effects on viral propagation were evaluated assuming that a difference of one cycle corresponds to a two-fold difference in the number of copies of viral RNA. Ct values obtained for drug-free samples were considered as 100 % viral propagation.

### 2.8 Evaluation of growth inhibition and toxicity of test drugs in non-infected mammalian cells

Effects of emetine and its analogs on cell growth were assessed by MTS and sulforhodamine assays. Detailed methods are provided in Supplementary Information.

### 2.9 Evaluation of effects on I_Ca-L_

#### 2.9.1 Dissociation of guinea pig ventricle

All procedures with guinea pigs were approved by the Stony Brook IACUC. Guinea pigs (500 g) were euthanized by sodium pentobarbitone overdose (1 mL of 390 mg/ml, *i.p*). Guinea pig ventricular myocytes were enzymatically isolated as described (Gao et al., 1992). Briefly, the heart was removed and cannulated *via* the aorta for backward perfusion with collagenase (Worthington Biochemical Co, NJ, USA). Following perfusion, a piece of ventricular tissue was removed and teased into small pieces in KB solution (Isenberg and Klockner, 1982) containing: 83 mM KCl, 30 mM K_2_HPO_4_, 5 mM MgSO_4_, 5 mM Na-pyruvic acid, 5 mM β-OH-butyric acid, 5 mM creatinine, 20 mM taurine, 10 mM glucose, 0.5 mM EGTA, 5 mM HEPES, 5 mM Na_2_-ATP (pH 7.2, adjusted with KOH). The ventricular tissue was triturated with an electric shaker for 1 min. The dissociated cells were kept in KB solution at 37 °C for ∼1 hour before the experiment.

#### 2.9.2 Human iPSC-derived ventricular cardiomyocytes

iPSC cells (Axol Bioscience Ltd, FL, USA) were plated at a density of 25,000 cells/cm^2^ on coverslips coated with 1x Axol Fibronectin Coating Solution in Axol Plating Medium (Axol Cardiomyocyte Maintenance Medium, 10 % fetal bovine serum and 10 μM Y-27,632 2HCl) according to manufacturer's instructions. The next day the medium was changed to Axol Cardiomyocyte Maintenance Medium. Cells were maintained at 5 % CO_2_, 37 °C in humidified conditions. Medium was replaced every other day.

#### 2.9.3 Patch clamp recording of I_Ca-L_ current in GP-ventricle and IPS-derived ventricular myocytes

An Axopatch −1D (Axon Instruments: Molecular Devices LLC, CA, USA) amplifier and Axopatch 10.3 software were used for recording and data analysis. Patch electrode resistance was set at 4–6 MΩ and room temperature was 22 ± 1 °C. During the recording, cells were held at −50 mV, then, the voltage was gradually raised to +60 mV in 10 mV increments with 300 ms pulse. The bath solution included the following components: 137 mM TEA-Cl, 1 mM MgCl_2_, 2 mM CaCl_2_, 10 mM HEPES, 10 mM glucose (pH 7.4, adjusted with TEA-OH). For the pipette the solution contained: 111 mM CsCl, 20 mM TEA-Cl, 10 mM glucose, 10 mM HEPES, 14 mM EGTA, 5 mM MgATP (pH 7.2, adjusted with CsOH). The I_Cal_ currents were recorded in the absence and presence of indicated concentrations of emetine or analog in three independent experiments for each drug.

### 2.10 Spectroscopic methods

#### 2.10.1 UV/Vis spectrophotometry

Absorbance profiles were obtained on an AVIV Biomedical Model 14 UV/Vis Spectrophotometer (Lakewood, NJ, USA) at 1.0 nm intervals over the wavelength range of 200 - 800 nm employing an averaging time of 5 s and slit width of 1 nm. Concentration-dependent measurements in ethanol (10 to 2500 µM range) were conducted to determine the propensity for intermolecular aggregation.

#### 2.10.2 Fluorescence properties

Fluorescence properties were characterized on an AVIV Biomedical Model 107 Differential Fluorescence Spectrophotometer (Lakewood, NJ, USA). Fluorescence emission spectra of emetine and its analogs were acquired in a 10 mm quartz cuvette at 1.0 nm intervals over the wavelength range of 250 - 600 nm employing an averaging time of 5 s and excitation/emission slits of 10 nm.

#### 2.10.3 Circular Dichroism spectroscopy

Chiroptical properties were characterized on an Aviv Model 420 Circular Dichroism Spectropolarimeter (Aviv Biomedical Inc., Lakewood, NJ) using a 1 mm quartz cuvette. CD spectra were acquired in a 1.0 mm quartz cuvette over the wavelength range of 220 - 350 nm at 1.0 nm resolution employing an averaging time of 30 s. The representative spectra are buffer subtracted, smoothed via the Savitzky-Golay algorithm, and concentration normalized to units of molar ellipticity.

### 2.11 Data analysis

Antiviral experiments performed with HCoV-OC43, host-cell toxicity assays, and protein synthesis experiments in lysates and cells involving emetine and DHE4 were repeated independently on different weeks at least three times, and 2–3 wells for each exposure condition within one experiment are reported as mean values with standard deviations. When experiments were conducted twice, results were reported as an average of two measurements. Two independent antiviral experiments were performed with SARS-CoV-2 with one experimental sample per condition. For compounds that showed little or no activity in these assays, two or three independent experiments were conducted to confirm the findings. Agreement between experiments was within 10–15 % from the average value. Four-parameter non-linear logistic regression fits were applied to evaluate 50 % inhibitory concentrations of investigated compounds. Total growth inhibition concentrations of drugs were obtained by manual evaluation of data collected for two-fold serial dilution of drugs, 12 concentrations in total. The difference between two conditions was considered as significant if the *p*-value was below 0.05 established by a two-sided Student's *t*-test.

Dissociation constants (*K_D_*) values for the inhibition of I_CaL_ by emetine, DHE and their stereoisomers were estimated using the following equation:

$$\%\;\text{inhibition}\;=\frac{D}{D+Kd}$$

D represents the concentrations of a given drug.

## 3 Results

### 3.1 Emetine and derivatives

We have studied two ipecac alkaloids of natural origin, emetine and its epimer isoemetine, as well as four diastereomers of synthetic dehydroemetine (DHE1, DHE2, DHE3 and DHE4) as described in Supplementary Table S1. The structures of emetine and its congeners are depicted in Supplementary Table S2. Emetine contains four chiral centers, in the 1R, 2S, 3R, 11bS-configuration. Due to unsaturation at C2-C3, DHE possesses only two chiral centers and four diasteriomers of which the 1R, 11bS conformer (DHE4) exhibits similar pharmacological properties to those of emetine. In principle, the basic physicochemical properties of emetine and analogs harbor a number of drug-like qualities that fulfill the requirements for bioavailable oral therapeutics (Lipinski, 2016) (Supplementary Table 3).

### 3.2 Emetine does not inhibit RNA-extension activity of RdRp

**Fig 2:**
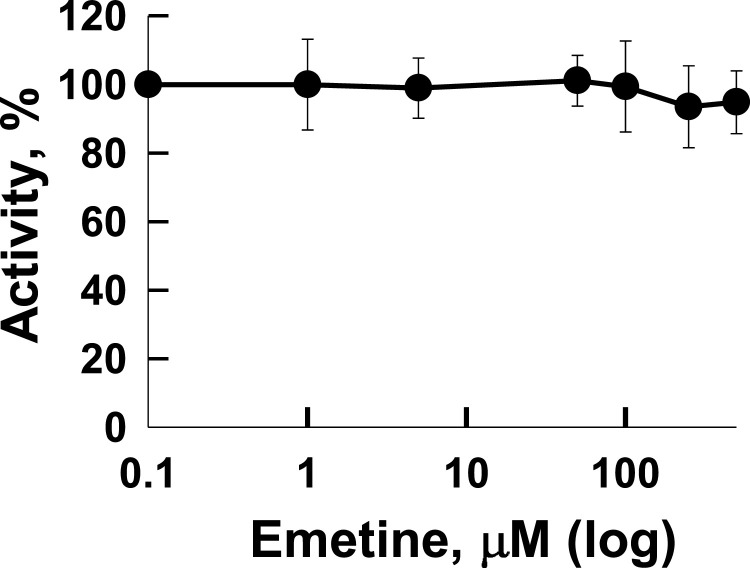
Activity of SARS-CoV-2 RdRp complex in the presence of emetine. In vitro polymerization activity of the RdRp in the presence of various emetine concentrations. The plot shows the mean (dots) and standard deviations (error bars) of at least 3 repeats for each emetine concentration.

Since several reports suggest that RdRp of coronaviruses might be inhibited by emetine, we evaluated the inhibitory activity of this compound on the SARS-CoV-2 RdRp complex. The nsp12–7–8 complex constitutes the essential RdRp core endowed with polymerization activity (Hillen et al., 2020). Recombinant nsp12 was expressed in insect cells while the nsp7 and nsp8 cofactors were expressed in *E. coli*. Following assembly of a functional complex, nsp12–7–8 exhibits RNA polymerization activity in RNA extension assays (Yin et al., 2023). Significantly, the addition of emetine elicits minimal or no inhibitory effect on polymerization activity of the RdRp complex at concentrations of 1 mM (Fig. 2) making SARS-CoV-2 RdRp an unlikely target for this class of compound.

### 3.3 Activities of emetine and derivatives as protein synthesis inhibitors: cell-free studies

**Fig. 3:**
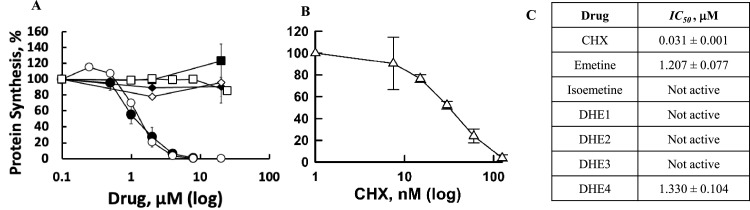
Structure-activity relationships of ipecac alkaloids as protein synthesis inhibitors in a cell free system. Ipecac alkaloids (A) or cycloheximide (B) were incubated with lysates of rabbit reticulocytes and mRNA coding for luciferase as described in Materials and Methods. The dose-dependent change in chemiluminescence of the translated luciferase is shown. Filled circles – emetine; empty circles – DHE4; filled squares – DHE2; empty squares – isoemetine; filled diamonds – DHE1; empty diamonds – DHE3. (C) shows mean and standard deviation values for the 50 % inhibitory concentrations (*IC_50_*) obtained from at least three independent experiments for each drug.

Another activity of emetine and its analogs that may account for antiviral activity is inhibition of protein synthesis. We tested the potential of these compounds to inhibit translation in rabbit reticulocyte lysate (RRL) using mRNA coding for firefly luciferase (Minetti et al., 2022). Emetine and DHE4, which share the 1R, 11bS configuration, are similar in protein synthesis inhibition, with IC_50_ values of 1.2 and 1.3 μM, respectively (Fig. 3A and C). DHE1, DHE2, DHE3 and isoemetine, exhibit no inhibitory effects over a broad concentration range (0.1 - 100 μM). The positive control, cycloheximide (CHX) inhibits protein synthesis with an IC_50_ of 31 nM (Fig. 3B and C). The data corroborate the reported structural requirement (Grollman, 1966b; Grollman, 1966a; Grollman, 1968; Grollman and Jarkovsky, 1975; Grollman, 1970) for ipecac alkaloids to inhibit protein synthesis, namely the 1R, 11bS-configurations.

### 3.4 Activities of emetine and derivatives as protein synthesis inhibitors: studies in cultured cells

We evaluated the effects of emetine and analogs on protein synthesis in two cell lines. Vero E6 is a monkey kidney epithelial cell line widely used in viral studies, while BEC-hACE2 is a human bronchial epithelial cell line that expresses the receptor for SARS-CoV-2 (hACE2) (Sohn et al., 2021). To monitor effects on protein synthesis we exposed cells to ipecac alkaloids for 1 h followed by 10 min of pulse labeling with puromycin. Puromycin is incorporated into the nascent protein chains and blocks further translation, thereby producing puromycilated peptides (Minetti et al., 2022; Dadehbeigi and Dickson, 2013). These peptides can be detected using standard immunoblotting techniques, employing anti-puromycin antibodies. Protein synthesis inhibitors reduce the amount of puromycilated proteins in a concentration-dependent manner.

**Fig. 4:**
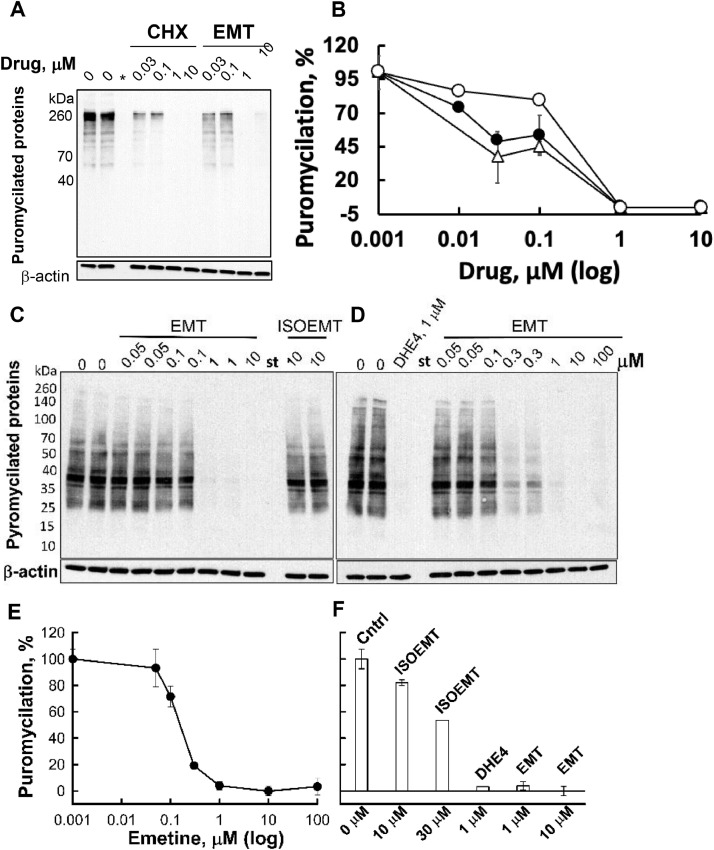
Protein synthesis in cultured Vero E6 (A, B) or BEC-hACE2 cells (C to F) in the presence of ipecac alkaloids. Cells were exposed to ipecac alkaloids or cycloheximide (CHX) for 1 h followed by pulse-labeling with puromycin, total protein isolation and immunoblotting as described in Materials and Methods. A, C, D – Representative immunoblots performed with anti-puromycin antibodies (top) or anti-actin antibodies (bottom). Concentrations of drugs used are shown above each lane. St - indicates the lane that contained only commercial size standards. B, E, F – Quantification of the puromycilation signal, normalized for β-actin, for varying doses of drugs. B – Vero E6 cells: CHX (empty triangles), emetine (filled circles) and DHE4 (empty circles). E – BEC-hACE2 cells treated with emetine. F – protein synthesis shown for representative concentrations of each drug. Quantitative data, normalized to actin expression, are presented as mean or average values collected from two to four plates of cells in two or three independent experiments. Where more than 2 plates were analyzed for selected concentrations, standard deviations are shown as well. When the standard deviation is not presented, the agreement between two experiments was within 20 % of the average value.

In both Vero E6 cells (Fig. 4A and B, Supplementary Fig. S3) and BEC-hACE2 cells (Fig. 4C-F) emetine and DHE4 inhibited protein synthesis with 1 µM drug terminating all translation activity (Fig. 4A and B). At 0.01 μM of emetine or DHE4, the protein synthesis is reduced 15 %−20 %. The positive control, CHX, exhibited greater activity and an analysis of the inhibition data in Vero E6 cells reveals that 50 % inhibitory activities of emetine, DHE4 and CHX are ∼ 0.11, 0.21 and 0.03 μM, respectively. Isoemetine, DHE1, DHE2, and DHE3 are inefficient as protein synthesis inhibitors.

It is interesting to note that the compounds evaluated herein failed to sustain inhibition of puromycilation in Vero E6 cells when employed at higher concentrations (i.e., > 10 μM for emetine and DHE4, Supplementary Fig. S3A; and > 1 μM for DHE1, DHE2, DHE3, and isoemetine, Supplementary Fig. S3B). While not fully understood, altered effects on puromycilation by emetine within the mid-high micromolar range has been reported (David et al., 2012; David et al., 2013). The enhancement of puromycilation rates within the mid-high micromolar range is independent of stereoisomeric conformation (Supplementary Fig. S3), implying that the effect is nonspecific and/or uncoupled from the stereoisomer-specific inhibition at lower concentrations.

Emetine inhibited protein synthesis in BEC-hACE2 cells by 30 %, 80 %, 97 % and 100 % when present at 0.1, 0.3, 1 and 10 μM, respectively (Fig. 4C–F). These measurements indicate an IC_50_ ∼ 0.12 μM. DHE4 inhibits protein synthesis by 97 % at 1 μM (Fig. 4B and F) a comparable value to emetine. At concentrations of 10 and 30 μM, isoemetine inhibited protein synthesis by 20 % and 50 %, respectively (Fig. 4A and F). In summary, the SAR of ipecac alkaloids in both a cell-free system and cultured cells are consistent, demonstrating that emetine and DHE4 are the most potent ipecac compounds in reducing protein synthesis in mammalian cells.

### 3.5 Antiviral activities and selectivity of emetine and derivatives

#### 3.5.1 HCoV-OC43

**Fig. 5:**
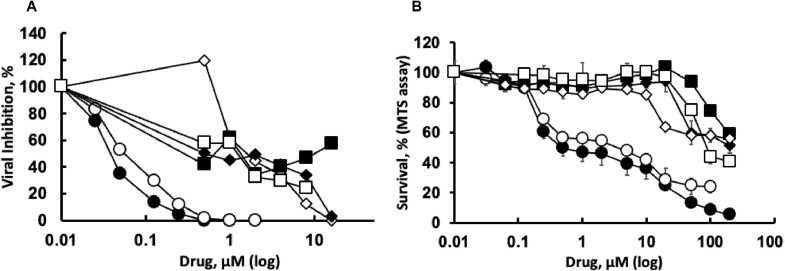
Antiviral activity and selectivity of ipecac alkaloids toward HCoV-OC43 virus in Vero E6 cells. A – Emetine, or an analog, was introduced after absorption of HCoV-OC43 (MOI 20). Viral RNA in the medium 72 h following infection was isolated and viral growth was evaluated by RT-qPCR for the N-gene. B - Non-infected Vero E6 cells were exposed to ipecac alkaloids for 72 h followed by MTS assay to evaluate growth inhibition and toxicity in response to drugs. Experimental details are described in Materials and Methods. Filled circles – emetine; empty circles – DHE4; filled squares – DHE2; empty squares – isoemetine; filled diamonds – DHE1; empty diamonds – DHE3.

Next, we address the inhibitory effects of ipecac alkaloids on the growth of HCoV-OC43, a mildly pathogenic beta coronavirus, employing Vero E6 cells. Viral replication is evaluated by RT-qPCR analysis of the viral *N*-gene present following incubation of infected cells with each compound for 72 h. Of the compounds studied, emetine and DHE4 displayed the highest antiviral activity with IC_50_ values of 40 and 63 nM, respectively (Fig. 5A, Table 1). Similar results are obtained for emetine 48 h post-infection (data not shown). Isoemetine, DHE1, DHE2 and DHE3 affected viral growth with IC_50_ values on the order of 1 - 2 μM (Fig. 5A, Table 1).

**Table 1:** Selectivity of ipecac alkaloids toward HCoV-OC43 virus in Vero E6 cells and their effects on protein synthesis in this cell line. Quantification of data as such shown in Figs. 4 and 5. Conditions when drugs were introduced for 1 h before viral absorption and present 72 h following infections are indicated as 1 hbi/72 hpi. Protein synthesis inhibition was identified by puromycin labeling and shown as a percent signal of puromycilated proteins in comparison with untreated control.

Compound	Antiviral IC_50_, μM	Fifty percent effect (*CC_50_*) in non-infected Vero E6, μM	Selectivity index, (*SI*) *CC_50_/IC_50_*	Protein synthesis,%
EMT (72 hpi)	0.040 ± 0.002	1.52 ± 0.41	38	0.01 μM: 20 % 0.03 μM: 50 % 0.1 μM: 50 % 1 μM: 100 % > 10 μM: signal increase
EMT (1 hbi/72 hpi)	0.044 ± 0.002	1.52 ± 0.41	35	
DHE4 (72 hpi)	0.063 ± 0.004	3.27 ± 0.83	52	0.01 μM: 15 % 0.1 μM: 20 % 1 μM: 100 % > 10 μM: signal increase
DHE4 (1 hbi/72 hpi)	0.066 ± 0.006	1.52 ± 0.41	35	
ISOEMT (72 hpi)	1.048 ± 0.213	114.3 ± 13.08	109	0.1 μM: 0 % >1 μM: signal increase
DHE1 (72 hpi)	1.027 ± 0.446	193.0 ± 63.58	188	
DHE2 (72 hpi)	2.309 ± 2.382	242.8 ± 50.44	105	
DHE3 (72 hpi)	2.028 ± 0.497	146.2 ± 51.91	72	

To establish selectivity indices (*SI*), we applied the MTS assay to evaluate metabolic activity of non-infected Vero E6 cells in response to various concentrations of the compounds (Fig. 5B). The cells in culture are at 70–80 % confluence when evaluated for antiviral and cytotoxicity effects. Since 48 h and 72 h of exposure to emetine and DHE4 produced similar results (data not shown), we focused on the 72 h time point. Dose response curves for emetine and DHE4 exhibited concentration-dependent decreases in MTS activity with an extended plateau, a range of concentrations with stable MTS activity (Fig. 5B). This behavior is characteristic of cytostatic drugs, as the plateau represents a concentration range at which drugs completely inhibit cell growth in the absence of direct cytotoxicity. Thus, emetine and DHE4 inhibit the growth of Vero E6 cells at low micromolar concentrations and are cytotoxic when present at concentrations higher than 10 μM. *SI* values are calculated by applying the drug concentrations needed to reduce the MTS signal by 50 % (*CC_50_*) in comparison to control cells, namely 1.52 and 3.27 μM μM for emetine and DHE4, respectively. Using these values, the *SI* for emetine and DHE4 are calculated as 38 and 52, respectively (Table 1). For the aforementioned reasons, the CC_50_ values obtained likely represent drug concentrations at which the growth of Vero E6 cells is fully inhibited, a phenomenon known as *TGI* (total growth inhibition). For the remaining ipecac alkaloids tested, the growth inhibition/toxicity curves yield CC_50_ values above 100 μM (Table 1, Fig. 5) and *SI* values ranging from ∼ 70 to 200. Collectively, our results reveal that emetine and DHE4 inhibited HCoV-OC43 growth (IC_50_ ∼ 40–60 nM) and host protein synthesis (IC_50_ ∼150–170 nM) in Vero E6 cells at nanomolar concentrations, suggesting that the antiviral effects of these drugs may be attributed to protein synthesis inhibition. Conversely, the remaining analogs exhibited greater *SI*s than emetine and DHE4 against HCoV-OC43, which infers that these compounds are not effective host protein synthesis inhibitors, and their rather modest antiviral action may result from unrelated mechanisms.

#### 3.5.2 SARS-CoV-2

**Fig. 6:**
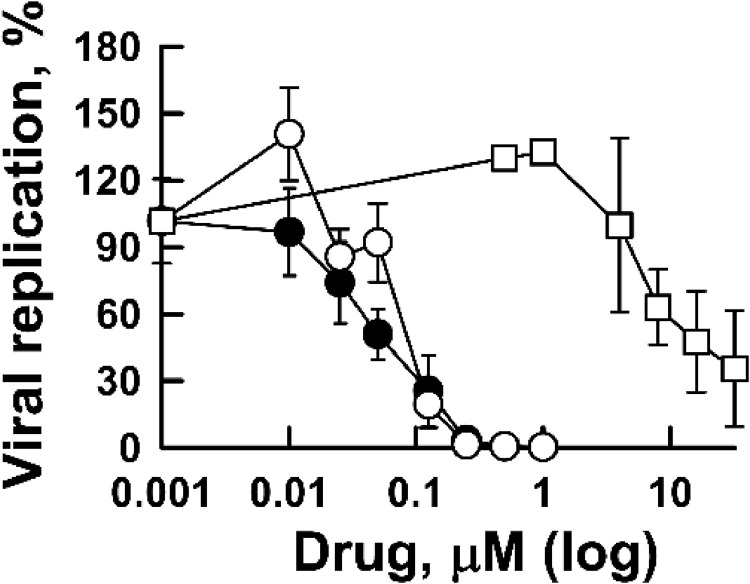
Antiviral activity of selected ipecac alkaloids against SARS-CoV-2 virus in BEC-hACE2 cells. BEC-hACE2 cells were infected with SARS-CoV-2 (0.01 pfu per cell) for 2 h followed by incubation with emetine (filled circles), DHE4 (empty circles) or isoemetine (empty squares) for 24 hpi. Viral RNA from media was isolated and viral propagation was evaluated by RT-qPCR. Experimental details are described in Materials and Methods.

We evaluated the antiviral effects and toxicities of emetine, isoemetine and DHE4 in human bronchiolar epithelial cells that express the SARS-CoV-2 receptor, BEC-hACE2, following infection with the SARS-CoV-2. In subconfluent BEC-hACE2 cells, emetine and DHE4 inhibited the growth of SARS-CoV-2 when added immediately following the viral absorption, with IC_50_ of 52 and 88 nM respectively at 24 hpi. Isoemetine also reduced the amount of viral RNA in the culture medium, albeit with a higher IC_50_ of 16.5 μM (Fig. 6, Table 2).

**Table 2:** Inhibitory concentrations of ipecac alkaloids in BEC-hACE2 cells and activity against SARS-CoV-2. CC_50_ – 50 % growth compared to control, TGI – total growth inhibition, GI_50_ – 50 % growth inhibition, IC_50_ – 50 % protein synthesis inhibition, MTS – 3-(4,5-dimethylthiazol-2-yl)-5-(3-carboxymethoxyphenyl)-2-(4-sulfophenyl)−2H-tetrazolium*,* SRB – sulforhodamine B.

Drug	MTS, CC_50_ μM (50 % effect)	MTS, *TGI* μM (beginning of the plateau)	SRB, GI_50_ μM	SRB, *TGI* μM	Protein synthesis inhibition,%	Anti-SARS-CoV2, IC_50_ μM
EMT	100 μM: cytostatic	0.125	0.112 ± 0.022	∼ 0.25–0.5	0.05 μM: 7 % 0.1 μM: 30 % 0.3 μM: 80 % 1 mM: 97 %	0.052 ± 0.003
DHE4	Effect not reached, 40 % decrease at 100 μM: cytostatic	0.125	0.062 ± 0.017	∼0.125–0.25	1 μM: 97 %	0.088 ± 0.023
ISOEMT	100 μM: cytotoxic	ND	19.44 ± 3.86	∼200 μM: toxic	10 μM: 18 % 30 μM: 47 %	16.48 ± 6.24

**Fig. 7:**
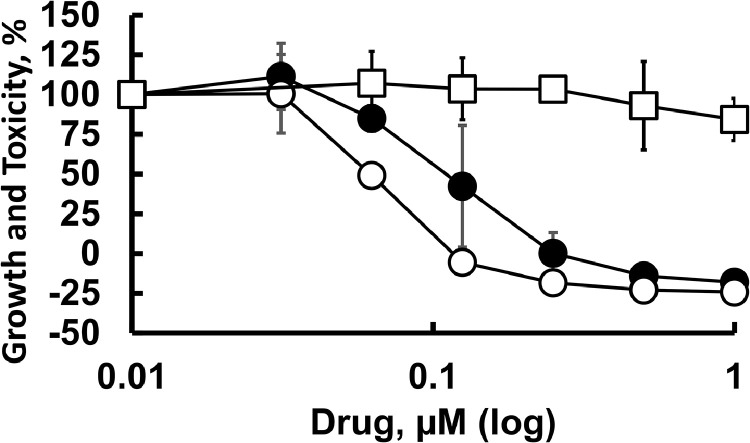
Cytostatic effects of ipecac alkaloids in BEC-hACE2 cells. Sub-confluent BEC-hACE2 cells were exposed to ipecac alkaloids for 24 h followed by SRB assay to evaluate growth inhibition and toxicity. Presented graph demonstrate changes in the SRB absorbance in response to varying concentrations of emetine, DHE4 and isoemetine. The growth of drug-treated cells at each drug concentration was determined using change in absorbance of the mock-treated cells between day 0 and 24 h as indicative of 100 % growth. All data are shown as mean values and standard deviations were obtained from three independent experiments. Filled circles – emetine; empty circles – DHE4; empty squares – isoemetine.

*SI* indices for BEC-hACE2 cells were established by conducting both MTS assays (Supplementary Fig. S4, Table 2) and sulforhodamine (SRB) staining assays (Fig. 7, Table 2) in non-infected sub-confluent BEC-hACE2 cells treated with emetine, DHE4 or isoemetine for 24 h. Similar to Vero E6 cells, the dose-response curves for emetine and DHE4 exhibited three distinct phases suggesting the cytostatic nature of these drugs at low micromolar concentrations (Supplementary Fig. S4, Table 2). Conversely, isoemetine affected the cells only when present at concentrations above 50 μM, with a dose-response curve consistent with cytotoxicity (Supplementary Fig. S4, Table 2). We also conducted the MTS assay with fully confluent BEC-hACE2 cells. No changes in the absorption signal of the MTS assay were observed at concentrations below 10 micromolar in treated cells compared to mock controls, supporting our hypothesis of the cytostatic effect of these compounds (Supplementary Fig. S4, Table 2) in the MTS assay.

The cytostatic nature of emetine is also supported by the results of sulforhodamine (SRB) staining in growing BEC-hACE2 cells. The SRB reagent binds cell proteins in the trichloroacetic acid (TCA) fixed wells yielding a magenta color that is proportional to the cell biomass. In the SRB assay (Fig. 7), toxic drugs produce a signal below zero on the Y axis, while cytostatic compounds reduce the signal to OD values that approximate those recorded for day 0 and remain unchanged across multiple doses of the drug. The latter behavior is characteristic of emetine and DHE4 in BEC-hACE2 cells (Fig. 7). GI_50_ represents the dose of a drug at which 50 % growth inhibition occurs. GI_50_ and *TGI* values are estimated as 0.11 μM and ∼0.25–0.5 μM for emetine, and 0.06 μM and ∼0.13–0.25 μM for DHE4, respectively (Table 2).

The cytostatic nature of emetine and DHE4 poses difficulty for selectivity estimations in BEC-hACE2 cells. Table 2 designates the relevant concentrations of emetine, DHE4 and isoemetine obtained from SRB, MTS, antiviral and protein inhibition assays. A 50 % effect in growing cells estimated by the MTS assay yields *SI* indices for emetine and DHE4 that exceed 1500. At the same time, *TGI* values for SRB and MTS assays yield an *SI* of ∼2–4 for each drug. For isoemetine, *SI* is six when using MTS CC_50_ and 12 when applying SRB *TGI*. In contrast, GI_50_ values result in the selectivity of 1–2 for each drug. When compared to effects on protein synthesis, it is clear that viral growth, protein synthesis and cell growth are inhibited in the same dose range of emetine. Therefore, one should distinguish the selectivity of emetine with respect to mechanism of action and potential safety in vivo. Since emetine does not kill host cells in the dose range that accounts for its antiviral effectivity, brief exposure to low doses of this drug in the clinic may prove beneficial in terms of suppressing viral infection without toxic side-effects.

### 3.6 Calcium channel inhibition studies

#### 3.6.1 Inhibition of the guinea pig l-type calcium channel by emetine and derivatives

**Fig. 8:**
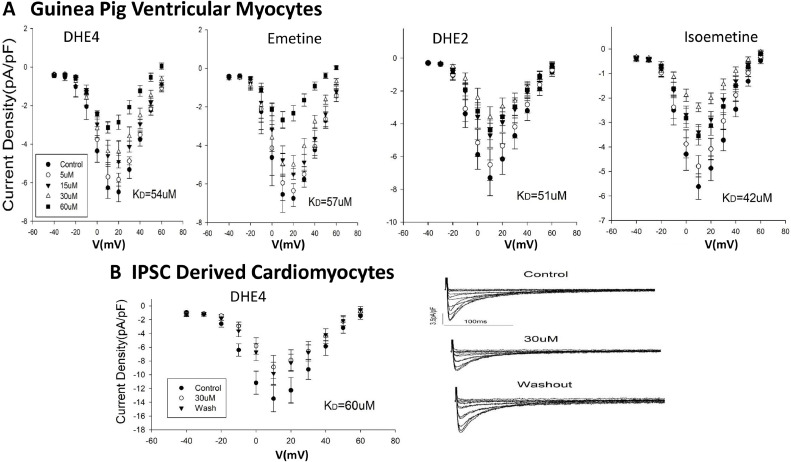
Effects of emetine and analogs on I_CaL_ in guinea pig ventricular myocytes and hIPS-derived cardiomyocytes (A) Peak I-V relationships in guinea pig ventricular myocytes from left to right panels are DHE4, emetine, DHE2, and isoemetine (B) Effect of DHE4 on I_CaL_ in human IPSC-derived cardiomyocytes: Left Panel shows peak current I/V relationship. The peak current densities are: 13.5, 8.9, and 9.8 for control, DHE4 (30 uM) and washout respectively. Filled circle-control, empty circle-DHE4 illustrate the individual current trace, under control, DHE4, filled triangle-washout. *N* = 6. The right panel illustrates the individual current traces under control, DHE4 and washout.

The clinical manifestation of emetine cardiotoxicity is reminiscent of established inhibitors of calcium channels I_CaL_. We therefore sought to determine the concentrations of emetine and analogs necessary to inhibit calcium channels I_CaL_ in guinea pig (GP) heart cells. To our knowledge, there is only one study that addressed the cardiotoxicity mechanisms of emetine (Lemmens-Gruber et al., 1996). Therefore, we applied the patch clamp technique to construct current-voltage (I-V) relationships for emetine and DHE4 to determine their inhibitory effects on I_CaL_ in GP cardiomyocytes. Four concentrations of each drug were evaluated (5, 15, 30 and 60 μM) in accordance with the previous study (Lemmens-Gruber et al., 1996). We find that both drugs affect I_CaL_ with similar degrees of inhibition: 57 μM *K_D_* for emetine and 54 μM *K_D_* for DHE4 when fit to the Langmuir binding isotherm assuming no cooperativity (Fig. 8A). Both DHE2 and isoemetine exhibit *K_D_* values similar to those of emetine and DHE4 (i.e., 42 and 50 μM for isoemetine and DHE2, respectively). Significantly, each emetine congener tested displayed *K_D_* values within the 40–60 μM range (data not shown), a clear indication that cardiotoxicity effects are unrelated to their properties as protein synthesis inhibitors.

#### 3.6.2 Inhibition of l-type calcium channel of human cardiomyocytes by DHE4

We confirmed that the effects found with GP cardiomyocytes apply to human cardiomyocytes by conducting patch clamp experiments employing hIPSC-derived cardiomyocytes. Since establishing and handling human cardiomyocytes in the current assay is more difficult than animal counterparts, the experimental design was limited to a single dose (30 μΜ) and one drug (DHE4). Inspection of Fig. 8B reveals a set of raw data for six repeats (*N* = 6). Applying the equation for a Langmuir binding isotherm, the mean *K_D_* for interactions between I_CaL_ and DHE4 is established as 60 μM. This estimate is consistent with the values derived in GP cardiomyocytes suggesting that there is no appreciable difference between species in response to these compounds with respect to inhibition of l-type calcium channel function in heart cells.

### 3.7 Optical properties of emetine and derivatives

**Fig. 9:**
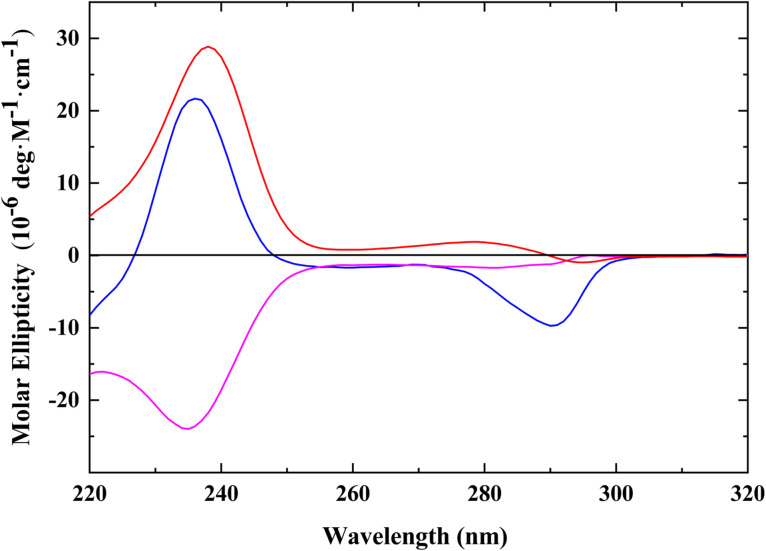
Chiroptical Profiles of Ipecac Alkaloids. Concentration-normalized circular dichroism spectra of emetine (blue), dehydroemetine (magenta), and isoemetine (red) expressed in units of molar ellipticity (For interpretation of the references to color in this figure legend, the reader is referred to the web version of this article.).

Emetine and DHE4 are unique among the molecules tested, by sharing the 1R, 11bS configurations, similar antiviral activities, and protein synthesis inhibition profiles. We conducted concentration-dependent spectroscopic analyses of these molecules (Supplementary Figs. S1A and S2A–C) without any evidence for self-aggregation (Supplementary Fig. S1A, inset). Moreover, the UV/Vis spectra of emetine and derivatives are virtually superimposable (data not shown). The fluorescence spectra of emetine (5 μM) excited at 280, 285, 290, and 295 nm are presented in Supplementary Fig. S1B. Emetine, isoemetine, and DHE4 exhibit similar fluorescence efficiencies; the presence of the C_2__—_C_3_ unsaturation in DHE does not appreciably impact the resultant spectra. We also examined the chiroptical properties of emetine and congeners via circular dichroism (CD) spectroscopy as illustrated in Fig. 9 and Supplementary Fig. S2A–C. Emetine exhibits a typical profile characterized by a negative Cotton effect at ∼ 288 nm and the presence of a positive Cotton effect at ∼ 237 nm, a pattern which has been associated with the *R,S* configuration of emetine at C-1 (Muhammad et al., 2003; Fujii et al., 1978). In contrast, isoemetine lacks a Cotton effect at ∼ 288 nm and exhibits a broader positive Cotton effect within the 270–280 region. These results are consistent with previous reports (Fujii et al., 1978) and confirm the identity and purity of both epimeric species (Muhammad et al., 2003; Fujii et al., 1978). Remarkably, the CD spectra of DHE4 exhibit significant differences in the region assigned for the C-1′ configuration relative to emetine (Fig. 9). Despite its similarity to emetine in terms of biological activity and absolute configuration (i.e. *R,S* configuration at C-1′), DHE4 exhibits a lower intensity negative ellipticity at ∼ 280 nm relative to emetine and a negative Cotton effect in the 230–240 nm region. These marked differences between emetine and DHE4 may be ascribed to the loss of 2 chiral centers due to the C2-C3 unsaturation.

## 4 Discussion

Based on their broad-spectrum activity against a wide range of infectious agents, it has been proposed that ipecac alkaloids should be considered for use in antiviral therapeutic regimens. Use of these drugs was discontinued due to adverse effects when given at exceedingly high doses to treat parasitic diseases. The clinically effective dose for treatment of amoebiasis was determined to be 1 mg of emetine or dehydroemetine per kg of body weight per day; 12 - 25 μM drug is required to inhibit the growth of *Entamoeba histolytica* in vitro (Burchard and Mirelman, 1988). The use of these drugs as anti-viral agents must therefore achieve a therapeutic window that is sufficiently below dose levels causing such adverse effects.

### 4.1 Emetine and DHE4 exhibit anticoronavirus activity at nanomolar concentrations in cultured cells

The current study reveals that DHE4 is the most potent synthetic congener of emetine and that emetine and DHE4 inhibit replication of SARS-CoV-2 in human bronchiolar cells in the 50–100 nM dose range. These IC_50_ values are consistent with the inhibitory activities of emetine and DHE4 against HCoV-OC43 in Vero E6 cells (40–60 nM), and with the potency of emetine against various coronaviruses reported by others as reviewed by Bleasel and Peterson (Bleasel and Peterson, 2020b). These concentrations are at least 125–250-fold lower than those required to kill amebae, suggesting that reducing the dose of ipecac alkaloids may afford protection against coronaviruses while avoiding undesirable side effects and toxicity.

### 4.2 Dose-dependent adverse effects and toxicity of ipecac alkaloids

We conducted electrophysiological studies of these compounds and their congeners to determine if the mechanisms underlying blocking of calcium channel current (I_CaL_) in cardiomyocytes would operate at the concentrations found to be effective against coronaviruses. Our interest in the calcium ion channel resides in reported clinical manifestations of emetine and dehydrometine cardiotoxicity at doses used in the past that are reminiscent of the effects observed for Diltiazem, a known blocker of I_CaL_ used for control of hypertension, which slows the heart rate and increases conduction time through the A-V node (Schwartz and Herrero, 1965; Kawai et al., 1981; Bergey and Much, 1986). Assuming 25 µM is the effective concentration of emetine in anti-ameba therapies, we estimate the reduction in the I_CaL_ in human cardiomyocytes is approximately one-third (Fig. 8B) and over thirty-three percent in GP ventricular myocytes (Fig. 8A). Our results reveal that emetine inhibits SARS-CoV-2 replication and protein synthesis in human epithelial bronchial cells with IC_50_ values of ∼ 52 and 200 nM, respectively. The emetine levels required to block I_CaL_ in GP cardiomyocytes are nearly 300-fold higher (i.e., IC_50_ ∼ 57 μM) than the antiviral *IC_50_*. Therefore, 0.5 μM emetine is sufficient to inhibit viral propagation and translation activity in host cells by at least 90 % yet only impacts I_CaL_ by 1 %, thereby resulting in minimal expected toxicity due to calcium channel inhibition. Even when applying a more conservative estimate of emetine activity against coronaviruses (0.5 μM *IC_50_*), 5 μM emetine would inhibit I_CaL_ by ∼10 % while remaining highly effective against SARS-CoV-2. Collectively, our results indicate that emetine cardiotoxicity due to I_CaL_ inhibition can be avoided by lowering the dose administered clinically to treat amebiasis by ten-fold while still retaining effective antiviral activity. Similar observations and conclusions apply to DHE4. Since emetine, dehydroemetine and their congeners exhibit comparable dissociation constants for the l-type calcium channel (i.e., range of 40 – 60 μM), inhibition of I_CaL_ is likely an off-target effect that is unrelated to the activities of these compounds as antivirals and/or protein synthesis inhibitors.

The diverse effects seen in patient EKGs indicate the involvement of other ion channels in the cardiotoxicity of ipecac alkaloids. Consequently, one might observe a duration prolongation of the QRS complex, which is associated with cardiac conduction via the sodium channel. However, a prior investigation has suggested a K_d_ of 15 μM for emetine-induced sodium channel reduction (Lemmens-Gruber et al., 1997), much higher than the concentration needed to achieve antiviral activity. Finally, besides investigating the effects of emetine and dehydroemetine on these, and other, ion channels, one must evaluate the concentration and rate-dependence as well as the autonomic responsiveness of action potentials from all cardiac regions. In this manner, each mechanism will be characterized by specific abnormalities that emerge in the EKG. However, when treated with low-dose emetine, administered subcutaneously at 1/10th of the dose adopted for amebiasis, effects are not observed in the electrocardiogram (EKG) of patients with viral hepatitis (Kosina and Truksova, 1976) or herpes zoster (Annamalai, 1965). Analysis of the resultant data will determine whether emetine dosages administered in the treatment of coronaviruses are completely free of any potential cardiac toxicity via ion channels that generate the action potential as well as its regulation by the autonomic nervous system.

### 4.3 Emetine and DHE4 antiviral activities are primarily attributed to host ribosomal protein synthesis inhibition

The antiparasitic activities of emetine and DHE are attributed to inhibition of protein synthesis (Entner and Grollman, 1973). Emetine and DHE interact with the E-site located in the 40S subunit of the *Plasmodium falciparum* ribosome and thereby inhibit peptide chain translocation (Wong et al., 2014; Dmitriev et al., 2020; Panwar et al., 2020). In contrast, there is minimal consensus on how these therapeutic agents exert their biological activity as antiviral drugs. Multiple inhibitory mechanisms have been proposed including viral cell entry (Shen et al., 2019; Yang et al., 2018), replication of viral genomes (Jan et al., 2021; Gurung et al., 2021), biogenesis of viral particles facilitated by host cell lysosomes (Choy et al., 2020; Yang et al., 2018), interactions between p53 and MDM2 controlled by mammalian RPS14 protein (Mukhopadhyay et al., 2016), and elongation of viral protein synthesis on mammalian ribosomes (Kumar et al., 2021). The suggested effect of emetine on SARS-CoV-2 RdRp is based on *in silico* modeling (Haider et al., 2022). To test whether emetine inhibits RdRp activity, we took advantage of a recently established experimental system to test effect on the activity of the recombinant catalytic complex of SARS-CoV-2 RdRp (Yin et al., 2023). We found that emetine concentrations as high as 1 mM did not reduce polymerase activity, suggesting that the inhibition of SARS-CoV-2 growth by emetine is not mediated in this way. In contrast, the inhibitory activity of both emetine and DHE4 against host protein synthesis was evident. While emetine is not as potent a protein synthesis inhibitor as cycloheximide, which we used as a positive control, it lacks other toxicities associated with cycloheximide such as DNA damage, teratogenesis, and disruption of spermatogenesis in animal models.

Due to the high selectivity indices (*SI*) of emetine against SARS-CoV-2 as gleaned from cell culture studies (i.e., ∼38–1000) (Choy et al., 2020; Bojkova et al., 2020; Jan et al., 2021; Wang et al., 2020a), investigations of its impact on the host cell have not been explored in antiviral studies. Significantly, the broad selectivity indices of emetine are not limited to SARS-CoV-2, but also are noted for other viruses including MERS and CMV (as reviewed by Bleasel) (Bleasel and Peterson, 2020b). Herein, we provide evidence that previously reported *SI*s for emetine are likely overestimated. It appears that the cytostatic effects of emetine in cells remained largely unnoticed. Interestingly, two reports claimed an anti-SARS-CoV-2 activity of emetine at low nanomolar and sub-nanomolar levels, leading to an estimation of its selectivity as 100 or even higher (Jan et al., 2021; Wang et al., 2020a). It is feasible that the high potency observed in several studies may be attributed to the use of indirect methods for evaluation of viral growth such as those employing antibodies against the SARS-CoV-2 NP protein (Jan et al., 2021). The broad range of emetine antiviral activities reported to date suggests that one or more host cell mechanism(s) may play a key role in the therapeutic efficacy of ipecac alkaloids. The therapeutic use of ipecac alkaloids to combat emerging infectious diseases represents a promising advancement in harnessing effective treatment strategies to improve our overall preparedness for future viral outbreaks.

### 4.4 Conclusions

Although emetine and dehydroemetine have been used in the clinic world-wide as anti-amebic agents their clinical use was marred by side-effects including cardiotoxicity and nausea at the doses required to treat amebiasis and this led to their replacement. Recent reports indicate that these alkaloids have anti-coronavirus activity including against SARS-CoV-2. We show that the anti-coronavirus activity of emetine, and the (-)-R,S isomer of dehydroemetine, occurs at two orders of magnitude less drug than that required for cardiotoxicity. Although there has been much speculation as to the antiviral mechanism of emetine, we show that inhibition of host-cell protein synthesis and inhibition of viral growth follow similar dose-response relationships and require the same stereochemistry.

## Funding

Authors would like to express deep gratitude to the Henry and Marsha Laufer Foundation and the Zickler Family Foundation for their long-standing philanthropic support. DPR is the recipient of The Collaborative Zickler Scholar in Translational Biomedical Research award. KJB acknowledges 10.13039/100007683NIH grants (GM23509 and GM34469) which over the years enabled the purchase and frequent upgrades of the state-of-the-art equipment used in the spectroscopic characterizations and assays reported herein.

## CRediT authorship contribution statement

**Viktoriya S. Sidorenko:** Writing – original draft, Visualization, Validation, Supervision, Project administration, Methodology, Investigation, Formal analysis, Conceptualization. **Ira Cohen:** Writing – review & editing, Supervision, Project administration, Methodology, Investigation, Formal analysis, Conceptualization. **Kunchok Dorjee:** Writing – review & editing, Conceptualization. **Conceição A. Minetti:** Writing – review & editing, Visualization, Investigation, Conceptualization. **David P. Remeta:** Writing – review & editing, Visualization, Investigation, Conceptualization. **Junyuan Gao:** Investigation. **Irina Potapova:** Investigation. **Hong Zhan Wang:** Investigation. **Janet Hearing:** Writing – review & editing, Visualization, Supervision, Investigation, Conceptualization. **Wan-Yi Yen:** Investigation, Conceptualization. **Hwan Keun Kim:** Investigation, Conceptualization. **Keiji Hashimoto:** Investigation. **Masaaki Moriya:** Investigation. **Kathleen G. Dickman:** Writing – review & editing. **Xingyu Yin:** Investigation. **Miguel Garcia-Diaz:** Writing – review & editing, Supervision, Investigation, Conceptualization. **Rajesh Chennamshetti:** Investigation. **Radha Bonala:** Investigation. **Francis Johnson:** Writing – review & editing, Supervision, Funding acquisition, Conceptualization. **Amanda L. Waldeck:** Writing – review & editing. **Ramesh Gupta:** Investigation. **Chaoping Li:** Investigation. **Kenneth J. Breslauer:** Writing – review & editing, Supervision, Funding acquisition, Conceptualization. **Arthur P. Grollman:** Writing – review & editing, Supervision, Funding acquisition, Conceptualization. **Thomas A. Rosenquist:** .

## Declaration of competing interest

The authors declare that they have no known competing financial interests or personal relationships that could have appeared to influence the work reported in this paper.

## Data Availability

Data will be made available on request.

## Appendix Supplementary materials

Image, application 1

Image, application 2

Image, application 3

Image, application 4

Image, application 5

Image, application 6

Image, application 7

Image, application 8
