# Supplementary material for: Mechanisms of antiviral action and toxicities of ipecac alkaloids: Emetine and dehydroemetine exhibit anti-coronaviral activities at non-cardiotoxic concentrations
Source: Virus Res. 2024 Jan 19;341:199322. doi: 10.1016/j.virusres.2024.199322 (PMC10831786; doi:10.1016/j.virusres.2024.199322)
Supplement: Supplementary file 1 [file mmc1.docx]

**Supplementary Methods**

***Inhibition of protein synthesis in cultured cells: puromycin pulse labelling***

Vero E6 cells were seeded at 30,000 cells per cm^2^ in 6-well plates. When cells achieved ~70-80% maximal density two days after plating, medium was replaced with 2% FBS-EMEM containing emetine or its analogs at 0.03-10 μM and plates were incubated for one hour followed by addition of puromycin (0.01 mg/ml final concentration). Puromycin labelling protocols have been well established by other laboratories and a general review of the methods has been published^79^. Control experiments were conducted in medium alone and medium containing cycloheximide or DMSO. Following incubation with puromycin for 12 min, the medium was quickly removed and cells were washed with warm PBS and then allowed to recover in drug-free 2% FBS-EMEM for 30 min. Cell pellets, collected by trypsinization and centrifugation for 5 min at 5 000 rpm at 4°C, were re-suspended in ice-cold PBS and the centrifugation was repeated. Collected cells were stored at -80°C until subjected to the protein preparation as noted below. Inhibition of protein synthesis in BEC-hACE2 cells by emetine and isoemetine was evaluated in a similar manner using standard culture medium for this cell line.

***Protein preparation and immunoblotting to detect puromycilated proteins***

Defrosted cell pellets were re-suspended in 120 μl of RIPA (radio-immunoprecipitation) buffer (Sigma; R0278) containing 7 μl of protease inhibitors (Sigma; P8340). Samples were incubated on ice for 40 min with occasional gentle mixing. Cell debris was removed by a 20 min centrifugation at 16,000 g at 4°C and supernatants were stored at -80°C. Protein was measured by the Pierce^TM^ bicinchoninic acid (BCA) assay (Thermo Fisher Scientific [TFS], MA, USA; 23250), using bovine serum albumin as a standard, according to protocols recommended by the manufacturer.

For immunoblotting, 10 μg of protein was combined with Laemmli buffer containing 10% β-mercaptoethanol, heated at 95°C for 5 min and loaded on 4-20% Mini-PROTEAN® TGX™ precast protein gels. Samples were resolved by electrophoresis in Tris-Gly-SDS buffer and transferred to nitrocellulose membranes. Transfer was conducted in Tris-Gly buffer containing 20% methanol at 4-6 V/cm^2^ for one hour. Membranes were blocked in 10 mM sodium phosphate buffer containing 150 mM NaCl (pH 7.8), 5% milk and 0.1% TWEEN20 (PBST) at room temperature for at least one hour, and then incubated overnight with primary anti-puromycin antibodies (TFS; MABE343 mouse monoclonal antibodies clone 12D10; 1:12,500) at 4°C. Membranes were washed for five minutes three times in PBST and incubated with secondary goat anti-mouse horseradish peroxidase (HRP) -conjugated antibodies (TFS, Invitrogen; G21040; 1:25,000) for 90 min. After three more rounds of washing as above, membranes were incubated with an HRP substrate (TFS; 34077) for one minute and chemoluminescence was visualized by exposing membranes to film for 1, 2, 5, 10 and 30 min.

For β-actin staining, the same membranes used for puromycin immunostaining were washed four times, 5 min each, in 20 mM Tris-buffered saline containing 500 mM sodium chloride (pH 7.5) and 0.1% TWEEN20 (TBST) and stripped by two rounds of 20 and 10 min washes at 50°C in glycine buffer (pH 2.2) containing 0.1 % SDS (W/V) and 1% TWEEN20. Membranes were then washed at room temperature with PBS (3x10 min) and PBST (2x5min). Blocking, primary (1:4,000) and secondary (1:5,000) antibody staining was conducted as above. Primary rabbit anti-β-actin antibodies were obtained from Cell Signaling (4967S, lot 9) and secondary HRP-conjugated goat-anti-rabbit antibodies were from the same company (7074S, lot 27).

Results were quantified with ImageJ 1.52a software using β-actin staining for each sample as an internal reference.

***Evaluation of growth inhibition and toxicity of test drugs in non-infected mammalian cells***

Vero E6 and BEC-hACE2 cells were grown to 70-80% density in their maintenance media and exposed to various concentrations of ipecac alkaloids (0.0125 – 200 μM) under standard culture conditions for 48-72 h and 24 h, respectively. Controls with varying amounts of DMSO and no DMSO were included also. Vero E6 cells were incubated with drugs in 2% FBS-EMEM, while BEC-HACE2 cells were grown and treated in complete PCS-300-03 medium with supplements. Similar treatments were also conducted using fully confluent non-growing BEC-hACE2 cells. At the end of the exposure period, MTS reagent (Promega G3580) was added at 1/5^th^ of the medium volume and color was allowed to develop by incubation of the plates for an hour in a cell culture incubator. To stop reactions, SDS was added to a final concentration of 2% and optical density of samples was measured at 450 or 490 nm, depending on the model of spectrophotometer (PharmaciaBiotech, NJ, USA; UV spectrophotometer, model Ultrospec2000) or plate reader (Molecular Devices, CA, USA; model Filter Max F5 Multi-mode microplate reader) available at the time of experiment.

Since BEC-hACE2 cells increased their metabolic activity in response to low doses of DMSO, thus complicating the evaluation of drug effects, we also conducted sulforhodamine B (SRB) assay to measure growth inhibition and toxicity by emetine in growing cells^82^. This method has been used by the National Cancer Institute to evaluate drug candidates for their activities as growth inhibitors and toxins in human cancer cell lines. NCI’s protocols for SRB assay were strictly followed^81, 82^. On the day of exposure (Day 0), several wells of untreated cells were fixed by addition of trichloroacetic acid (TCA, 10% V/V) and incubated for one hour at 4°C. The supernatant was discarded, wells washed five times with tap water and then air-dried. Samples were stored at room temperature until cells exposed to ipecac alkaloids or DMSO for 24 h were fixed in a similar manner. For color development, 500 μl of 0.4% SRB reagent prepared in 1% acetic acid was added to fixed cells and samples were incubated at room temperature for 15 min with gentle rocking. Unbound dye was removed by five washes with 1% acetic acid and plates were air-dried. Trizma base (10 mM) was used to dissolve the stain and absorbance was measured at 515 nm using a spectrophotometer. By comparing absorption values obtained at day 0 to the signal at day 1, with and without drugs, this protocol permits distinguishing growth inhibition from toxicity of the test compounds.
