# Supplementary material for: Mechanisms of antiviral action and toxicities of ipecac alkaloids: Emetine and dehydroemetine exhibit anti-coronaviral activities at non-cardiotoxic concentrations
Source: Virus Res. 2024 Jan 19;341:199322. doi: 10.1016/j.virusres.2024.199322 (PMC10831786; doi:10.1016/j.virusres.2024.199322)
Supplement: Supplementary file 2 [file mmc2.pptx]

## Slide 1
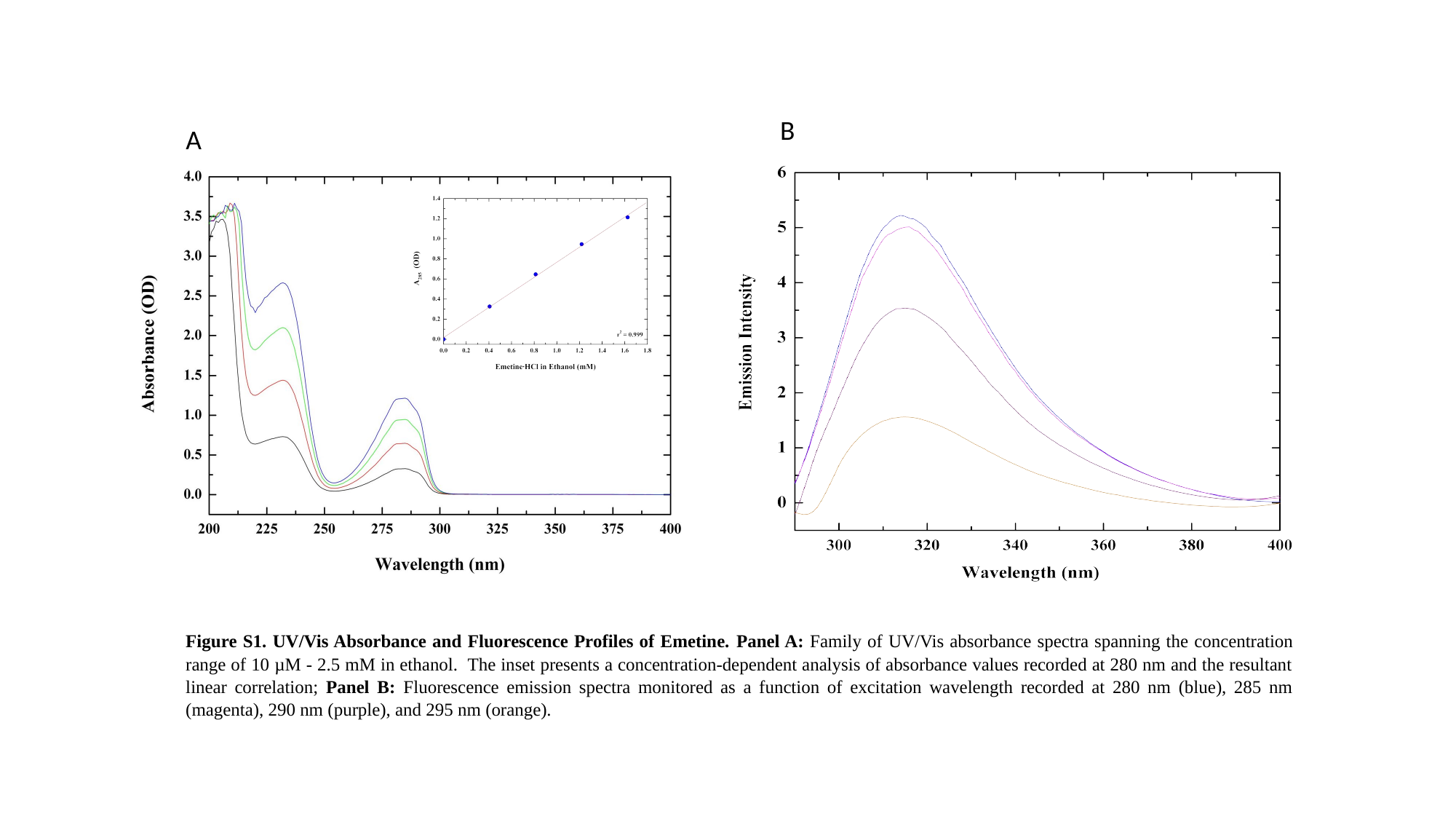

B
A
Figure S1. UV/Vis Absorbance and Fluorescence Profiles of Emetine. Panel A: Family of UV/Vis absorbance spectra spanning the concentration range of 10 µM - 2.5 mM in ethanol. The inset presents a concentration-dependent analysis of absorbance values recorded at 280 nm and the resultant linear correlation; Panel B: Fluorescence emission spectra monitored as a function of excitation wavelength recorded at 280 nm (blue), 285 nm (magenta), 290 nm (purple), and 295 nm (orange).
