# Supplementary material for: Mechanisms of antiviral action and toxicities of ipecac alkaloids: Emetine and dehydroemetine exhibit anti-coronaviral activities at non-cardiotoxic concentrations
Source: Virus Res. 2024 Jan 19;341:199322. doi: 10.1016/j.virusres.2024.199322 (PMC10831786; doi:10.1016/j.virusres.2024.199322)
Supplement: Supplementary file 3 [file mmc3.pptx]

## Slide 1
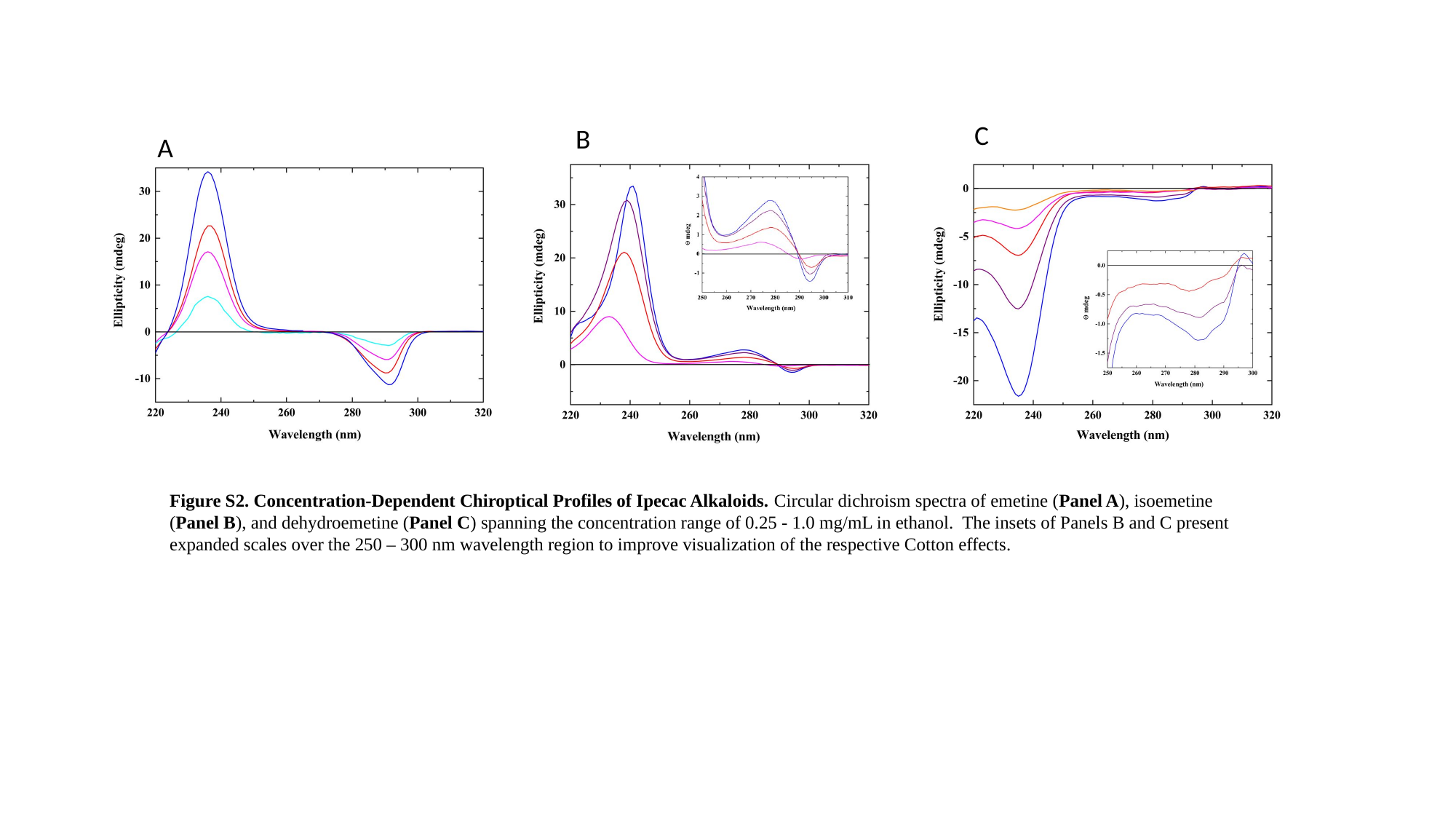

C
B
A
Figure S2. Concentration-Dependent Chiroptical Profiles of Ipecac Alkaloids. Circular dichroism spectra of emetine (Panel A), isoemetine (Panel B), and dehydroemetine (Panel C) spanning the concentration range of 0.25 - 1.0 mg/mL in ethanol. The insets of Panels B and C present expanded scales over the 250 – 300 nm wavelength region to improve visualization of the respective Cotton effects.
