# Supplementary material for: Mechanisms of antiviral action and toxicities of ipecac alkaloids: Emetine and dehydroemetine exhibit anti-coronaviral activities at non-cardiotoxic concentrations
Source: Virus Res. 2024 Jan 19;341:199322. doi: 10.1016/j.virusres.2024.199322 (PMC10831786; doi:10.1016/j.virusres.2024.199322)
Supplement: Supplementary file 4 [file mmc4.pptx]

## Slide 1
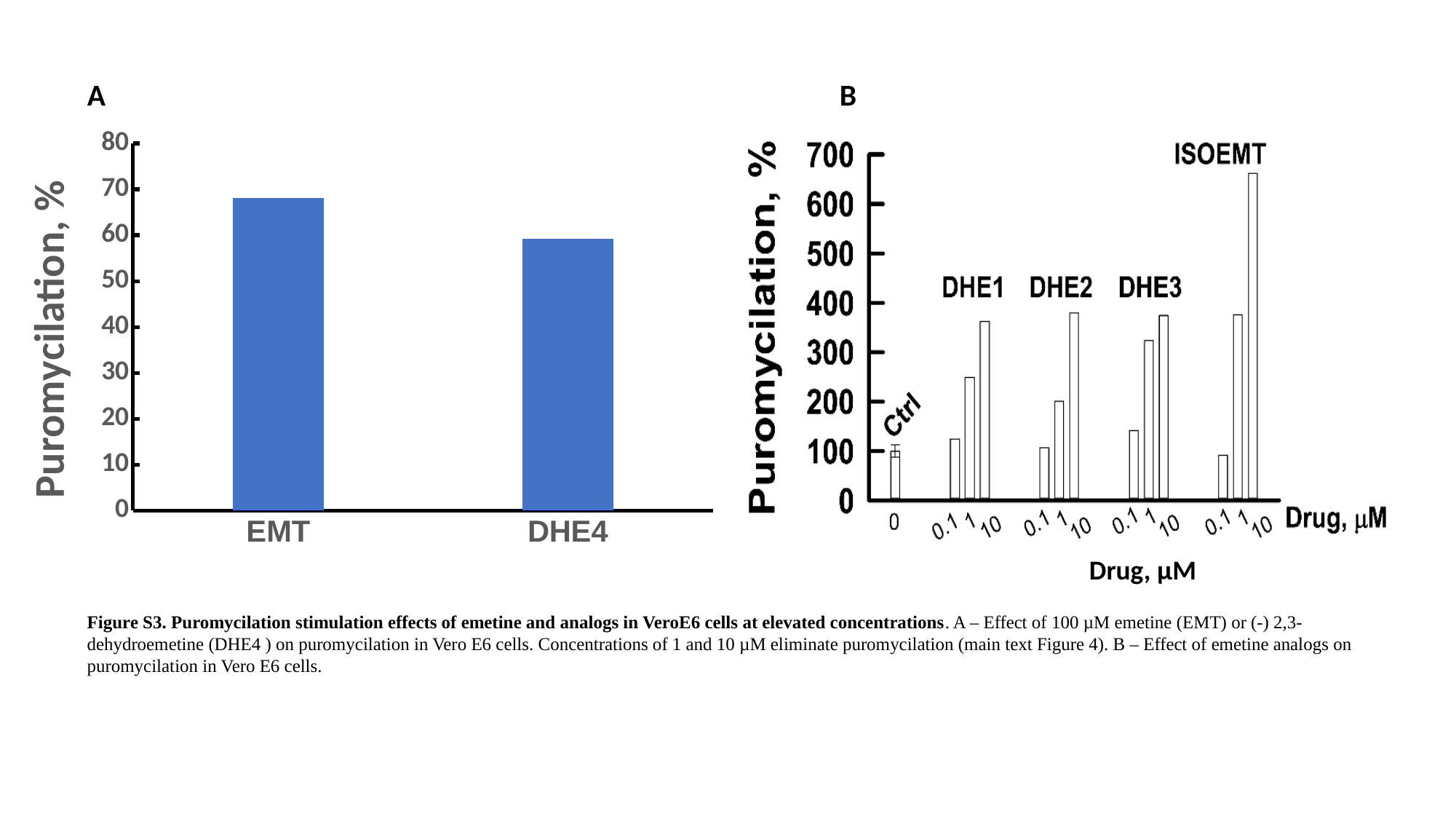

A
B
### Chart
| Category | average % |
|---|---|
| EMT | 68.15841118914304 |
| DHE4 | 59.13897202104855 |
Drug, µM
Figure S3. Puromycilation stimulation effects of emetine and analogs in VeroE6 cells at elevated concentrations. A – Effect of 100 µM emetine (EMT) or (-) 2,3-dehydroemetine (DHE4 ) on puromycilation in Vero E6 cells. Concentrations of 1 and 10 µM eliminate puromycilation (main text Figure 4). B – Effect of emetine analogs on puromycilation in Vero E6 cells.
