# Supplementary material for: Mechanisms of antiviral action and toxicities of ipecac alkaloids: Emetine and dehydroemetine exhibit anti-coronaviral activities at non-cardiotoxic concentrations
Source: Virus Res. 2024 Jan 19;341:199322. doi: 10.1016/j.virusres.2024.199322 (PMC10831786; doi:10.1016/j.virusres.2024.199322)
Supplement: Supplementary file 5 [file mmc5.pptx]

## Slide 1
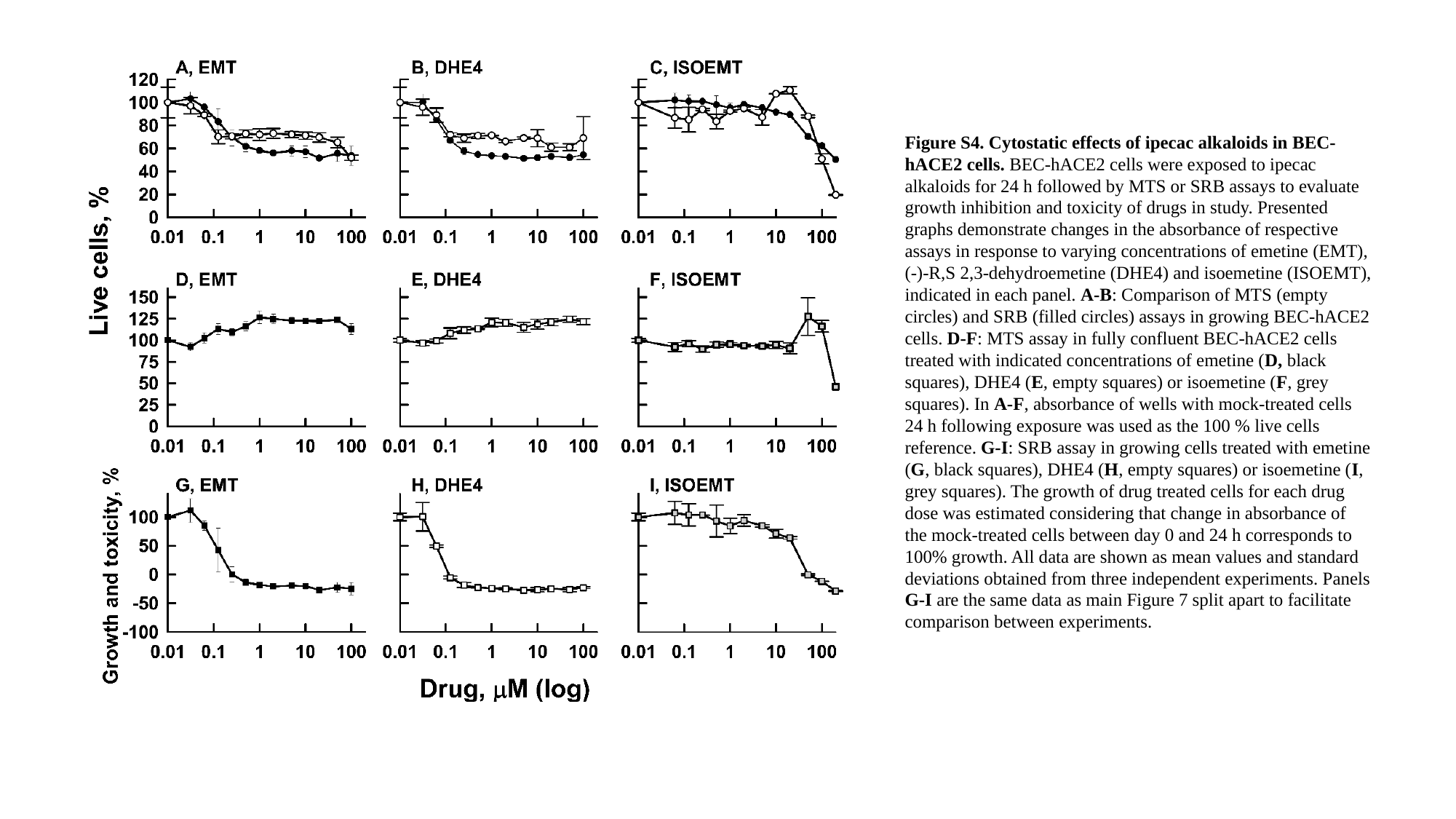

Figure S4. Cytostatic effects of ipecac alkaloids in BEC-hACE2 cells. BEC-hACE2 cells were exposed to ipecac alkaloids for 24 h followed by MTS or SRB assays to evaluate growth inhibition and toxicity of drugs in study. Presented graphs demonstrate changes in the absorbance of respective assays in response to varying concentrations of emetine (EMT), (-)-R,S 2,3-dehydroemetine (DHE4) and isoemetine (ISOEMT), indicated in each panel. A-B: Comparison of MTS (empty circles) and SRB (filled circles) assays in growing BEC-hACE2 cells. D-F: MTS assay in fully confluent BEC-hACE2 cells treated with indicated concentrations of emetine (D, black squares), DHE4 (E, empty squares) or isoemetine (F, grey squares). In A-F, absorbance of wells with mock-treated cells 24 h following exposure was used as the 100 % live cells reference. G-I: SRB assay in growing cells treated with emetine (G, black squares), DHE4 (H, empty squares) or isoemetine (I, grey squares). The growth of drug treated cells for each drug dose was estimated considering that change in absorbance of the mock-treated cells between day 0 and 24 h corresponds to 100% growth. All data are shown as mean values and standard deviations obtained from three independent experiments. Panels G-I are the same data as main Figure 7 split apart to facilitate comparison between experiments.
