# Supplementary material for: Mechanisms of antiviral action and toxicities of ipecac alkaloids: Emetine and dehydroemetine exhibit anti-coronaviral activities at non-cardiotoxic concentrations
Source: Virus Res. 2024 Jan 19;341:199322. doi: 10.1016/j.virusres.2024.199322 (PMC10831786; doi:10.1016/j.virusres.2024.199322)
Supplement: Supplementary file 6 [file mmc6.docx]

| **Sample ID** | **Full nomenclature** | **Source and Identity of Compounds** | **CAS ID** | **Other Notes** |
| --- | --- | --- | --- | --- |
| Emetine dihydrochloride (EMT, purity >98%) | 2H-Benzoaquinolizine, 3-ethyl-1,3,4,6,7,11b-hexahydro-9,10-dimethoxy-2-(1R)-1,2,3,4-tetrahydro-6,7-dimethoxy-1-isoquinolinylmethyl-, dihydrochloride, (2S,3R,11bS)- | Sigma Millipore Calbiochem: isolated from the ground roots of *Uragoga ipecacuanha* | 316-42-7 | Emetine is produced by plants mostly in the form of 1R,2S,3R,11bS. |
| Isoemetine dihydrochloride (ISOEMT, purity >98%) | 2H-Benzoaquinolizine, 3-ethyl-1,3,4,6,7,11b-hexahydro-9,10-dimethoxy-2-(1S)-1,2,3,4-tetrahydro-6,7-dimethoxy-1-isoquinolinylmethyl-, dihydrochloride, (2S,3R,11bS)- | ChemMaster: synthetic conversion from emetine-Calbiochem | ND | Stereospecific conversion of the natural emetine allowed to obtain pure isoemetine 1S,2S,3R,11bS |
| Dehydroemetine isomer 1 (DHE1)  (purity ~90%) | 4H-Benzo[a]quinolizine, 3-ethyl-1,6,7,11b-tetrahydro-9,10-dimethoxy-2-[[(1S)-1,2,3,4-tetrahydro-6,7-dimethoxy-1-isoquinolinyl]methyl]-, (11bR)- | MedChemExpress: full synthesis of dehydroemetine, isolated as one of the HPLC peaks, #1-4 by the order of appearance in the chromatogram using chiral sorbent. The identity of DHE4 as active dehydroemetine was assigned due to its activity as a protein synthesis inhibitor in cultured cells and cell free extracts. DHE1 is the mirror image of DHE4 based on the high resolution NMR data.  DHE2 and DHE3 are dehydroisoemetines: however, which one is 1S, 11bS- and 1R,11bR has not been determined. | 68831-62-9 | Mirror image of active dehydroemetine, DHE4 |
| Dehydroemetine isomer 2 (DHE2)  (purity >98%) | 4H-Benzo[a]quinolizine, 3-ethyl-1,6,7,11b-tetrahydro-9,10-dimethoxy-2-[[(1S)-1,2,3,4-tetrahydro-6,7-dimethoxy-1-isoquinolinyl]methyl]-, (11bS)-  AND  4H-Benzo[a]quinolizine, 3-ethyl-1,6,7,11b-tetrahydro-9,10-dimethoxy-2-[[(1R)-1,2,3,4-tetrahydro-6,7-dimethoxy-1-isoquinolinyl]methyl]-, (11bR)- |  | ND | 1S, 11bS dehydroisoemetine (DHE2 OR DHE3) is considered as an inactive component of the dehydroemetine sample used in clinic against amebiasis |
| Dehydroemetine isomer 3 (DHE3)  (purity ~90%) |  |  |  |  |
| Dehydroemetine isomer 4 (DHE4)  (purity >98%) | 4H-Benzo[a]quinolizine, 3-ethyl-1,6,7,11b-tetrahydro-9,10-dimethoxy-2-[[(1R)-1,2,3,4-tetrahydro-6,7-dimethoxy-1-isoquinolinyl]methyl]-, (11bS)- |  | 4914-30-1 | An active ingredient of the clinical formulation of dehydroemetine. Also known as 2,3 (-) dehydroemetine or dehydroemetine |

**Table S1. The sources and the IUPAC nomenclature of natural emetine and its synthetic analogs. In red and blue, highlighted atoms that define whether emetine and dehydroemetine are active or inactive, respectively, as protein synthesis inhibitors. ND: not determined**
