# Supplementary material for: Mechanisms of antiviral action and toxicities of ipecac alkaloids: Emetine and dehydroemetine exhibit anti-coronaviral activities at non-cardiotoxic concentrations
Source: Virus Res. 2024 Jan 19;341:199322. doi: 10.1016/j.virusres.2024.199322 (PMC10831786; doi:10.1016/j.virusres.2024.199322)
Supplement: Supplementary file 7 [file mmc7.docx]

| **Common accepted compound ID** | **Full nomenclature** | **Structure** |
| --- | --- | --- |
| Emetine | 2H-Benzoaquinolizine, 3-ethyl-1,3,4,6,7,11b-hexahydro-9,10-dimethoxy-2-(1R)-1,2,3,4-tetrahydro-6,7-dimethoxy-1-isoquinolinylmethyl-, dihydrochloride, (2S,3R,11bS)- |  |
| Isoemetine | 2H-Benzoaquinolizine, 3-ethyl-1,3,4,6,7,11b-hexahydro-9,10-dimethoxy-2-(1S)-1,2,3,4-tetrahydro-6,7-dimethoxy-1-isoquinolinylmethyl-, dihydrochloride, (2S,3R,11bS)- |  |
| (1-R, 11b-S)-2,3-dehydroemetine (DHE4 in MCE set, **Supplementary Table S1**) | 4H-Benzo[a]quinolizine, 3-ethyl-1,6,7,11b-tetrahydro-9,10-dimethoxy-2-[[(1R)-1,2,3,4-tetrahydro-6,7-dimethoxy-1-isoquinolinyl]methyl]-, (11bS)- |  |
|  (1-S, 11b-S)-2,3-dehydroisoemetine (DHE2 or DHE3 in MCE set, **Supplementary Table S1**) | 4H-Benzo[a]quinolizine, 3-ethyl-1,6,7,11b-tetrahydro-9,10-dimethoxy-2-[[(1S)-1,2,3,4-tetrahydro-6,7-dimethoxy-1-isoquinolinyl]methyl]-, (11bS)- |  |
| (1-R, 11b-R)-2,3- dehydroisoemetine (DHE2 or DHE3 in MCE set, **Supplementary Table S1**) | 4H-Benzo[a]quinolizine, 3-ethyl-1,6,7,11b-tetrahydro-9,10-dimethoxy-2-[[(1R)-1,2,3,4-tetrahydro-6,7-dimethoxy-1-isoquinolinyl]methyl]-, (11bR)- |  |
| (1-S, 11b-R)-2,3-dehydroemetine (DHE1 in MCE set, **Supplementary Table S1**) | 4H-Benzo[a]quinolizine, 3-ethyl-1,6,7,11b-tetrahydro-9,10-dimethoxy-2-[[(1S)-1,2,3,4-tetrahydro-6,7-dimethoxy-1-isoquinolinyl]methyl]-, (11bR)- |  |

**Table S2. Structures of natural emetine and its synthetic analogs**
