# Supplementary material for: Mechanisms of antiviral action and toxicities of ipecac alkaloids: Emetine and dehydroemetine exhibit anti-coronaviral activities at non-cardiotoxic concentrations
Source: Virus Res. 2024 Jan 19;341:199322. doi: 10.1016/j.virusres.2024.199322 (PMC10831786; doi:10.1016/j.virusres.2024.199322)
Supplement: Supplementary file 8 [file mmc8.docx]

| **PROPERTY** | **EMETINE** | **DEHYDROEMETINE** | **ISOEMETINE** | **Rule of 5** | **Other Criteria** |
| --- | --- | --- | --- | --- | --- |
| Molecular Weight | 480.6 | 478.6 | 480.6 | < 500 |  |
| XLogP3-AA | 4.7 | 3.7 | 4.7 | < 5 |  |
| AlogP | 4.9 |  |  | < 5 |  |
| Hydrogen Bond Donor Count | 1 | 1 | 1 | < 5 |  |
| Hydrogen Bond Acceptor Count | 6 | 6 | 6 | < 10 |  |
| HBD+HBA | 7 | 7 | 7 |  | < 10 |
| Rotatable Bond Count | 7 | 7 | 7 | < 10 |  |
| Exact Mass | 480.3 | 478.3 | 480.3 |  |  |
| Monoisotopic Mass | 480.3 | 478.3 | 480.3 |  |  |
| Topological Polar Surface Area | 52.2 Å² | 52.2 Å² | 52.2 Å² |  | < 140 |
| Heavy Atom Count | 35 | 35 | 35 |  |  |
| Complexity | 679 | 744 | 679 |  |  |
| Defined Atom Stereocenter Count | 4 | 2 | 4 |  |  |

**Table S3. Drug-like physical properties of emetine and analogs.**
